# Maternal lipidomic signatures of preterm and small-for-gestational-age newborn infants in low- and middle-income countries

**DOI:** 10.1126/sciadv.adu9145

**Published:** 2025-12-03

**Authors:** Ivana Marić, Ali Mahzarnia, Hilda A. Mujuru, Gwendoline Chimhini, Samir K. Saha, Mohammad Shameem Hassan, Nancy A. Otieno, Steven Hawken, Kumanan Wilson, Xiaotao Shen, Samuel Lancaster, Ronald J. Wong, Jonathan D. Reiss, John Kerner, Michael P. Snyder, William Hay, Gary M. Shaw, David K. Stevenson, Victoria Ward, Gary L. Darmstadt

**Affiliations:** ^1^ Department of Pediatrics, Stanford University School of Medicine, Stanford, CA, USA.; ^2^ University of Zimbabwe, Harare, Zimbabwe.; ^3^ Child Health Research Foundation, Dhaka, Bangladesh.; ^4^ Kenya Medical Research Institute, Nairobi, Kenya.; ^5^ Ottawa Hospital Research Institute and University of Ottawa, Ottawa, Canada.; ^6^ Bruyère Health Research Institute and Department of Medicine, University of Ottawa, Ottawa, Canada.; ^7^ Singapore Phenome Center, Lee Kong Chian School of Medicine, Nanyang Technological University; ^8^ School of Chemistry, Chemical Engineering and Biotechnology, Nanyang Technological University, Singapore, Singapore.; ^9^ Department of Genetics, Stanford University School of Medicine, Palo Alto, CA, USA.; ^10^ University of Colorado, Denver, CO, USA.

## Abstract

Maternal lipid levels change dynamically during gestation to support normal fetal growth. To obtain a detailed footprint of these changes and their differences in pregnancies with preterm or small-for-gestational-age (SGA) neonates, we analyzed 641 lipids and 639 metabolites in plasma from women by 24 weeks of pregnancy from three cohorts from low- and middle-income countries: Bangladesh, Zimbabwe, and Kenya. We consistently found a significant lipid imbalance with increased lipid levels that preceded preterm birth and decreased levels that preceded SGA births. Changes were most pronounced in triglycerides, including triglycerides containing proinflammatory omega-6 polyunsaturated fatty acids (PUFAs) in pregnancies with preterm infants. A machine learning model for prediction of preterm birth had modest performance [area under the receiver operator curve (AUC) = 0.69, 95% confidence interval (CI) = (0.68, 0.70)] and lower performance for predicting SGA [AUC = 0.64, CI 95% = (0.62, 0.65)]. Increased triglycerides containing proinflammatory PUFAs provide further evidence in favor of a previously considered dietary supplementation with the long-chain fatty acids.

## Teaser

Imbalance of early- and midpregnancy maternal lipids occurs in pregnancies with preterm and small-for-gestational-age infants.

## INTRODUCTION

Appropriate levels of maternal lipids have an essential role in maintaining a healthy pregnancy and normal fetal growth. These levels change markedly throughout gestation in two phases. The anabolic phase occurs during the first two trimesters and is characterized by an accumulation of triglycerides (TGs), FAs, and cholesterols in the blood (*1*). The increased lipogenesis and reduced lipolysis that occur in the anabolic phase are associated with increased maternal blood lipid levels (*2*). The second, catabolic phase, occurs in the third trimester and is characterized by an upregulation of lipolysis resulting in the breakdown of fat deposits and release of lipids necessary to support the growing fetus (*2*). Identifying differences from normal trends in maternal lipidomic dynamics might be predictive of slower fetal growth and pregnancy outcomes, including the birth of preterm and small-for-gestational-age (SGA) newborns. The lipidome includes a range of lipid-based compounds. The metabolome, more broadly, consists of a comprehensive set of small molecules produced by metabolism or from the environment (xenobiotics) that can be measured in a biological sample. The metabolome includes lipids among its main classes, together with amino acids, carbohydrates, nucleotides, coenzymes, and cofactors (*3*). Similar to the lipidome, metabolomic levels change throughout pregnancy (*4*, *5*). Establishing specific lipidomic and metabolomic biomarkers early and midpregnancy that are associated with risks of preterm birth (PTB) and SGA could be used for early prediction of these risks, elucidate the involved molecular pathways including alterations in lipids, and guide possible mitigating treatment strategies.

Low- and middle-income countries (LMICs) are disproportionately affected by a high prevalence of SGA infants, in large part due to higher rates of poverty and maternal undernutrition: 27% of infants in LMICs are SGA compared with 11% in high-income countries (*6*). Moreover, 81% of the 13.4 million PTBs globally each year occur in LMICs; rates are highest in Southern Asian (13.4%) and sub-Saharan African (10.1%) regions (*7*). Neonates born preterm or SGA are at increased risk for neonatal death (*8*) and a range of morbidities including neurodevelopmental disorders, stunted growth, and chronic diseases (*9*). Infants that are born preterm and/or are SGA have recently been classified as “small vulnerable newborns,” emphasizing the need to identify, characterize, and appropriately manage these conditions (*10*).

We performed a systematic analysis of 641 lipids and 639 metabolites measured midpregnancy in maternal plasma in three cohorts totaling 1151 women from LMICs. Our goal was to systematically investigate, using statistical and machine learning methods, whether changes in maternal lipidomics/metabolomics precede PTB and SGA outcomes; whether omics signatures of these outcomes can be identified, thereby facilitating midpregnancy prediction of PTB and SGA; whether these signatures generalize across cohorts; and whether the maternal metabolome influences neonatal metabolome at birth. Identification of specific lipids and metabolites altered in PTB and SGA pregnancies might provide an early prediction tool, offer insights into these outcomes, and guide possible treatments.

## RESULTS

### Study population characteristics

**Fig. 1.**
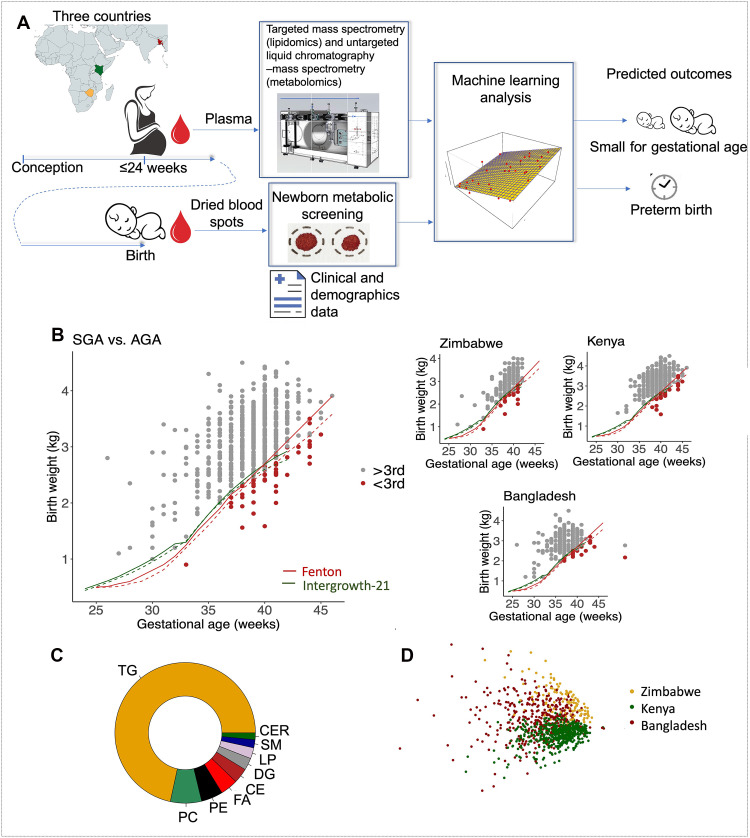
Study design. (**A**) Maternal plasma samples and clinical and demographic data were collected from 1390 mothers during pregnancy and analyzed using targeted mass spectrometry (MS; lipidomics) and untargeted liquid chromatography–MS (LC-MS; metabolomics). Associations of lipidomics and metabolomics data with PTB and SGA were obtained using univariate statistical analysis. Prediction models for PTB and SGA from these data were obtained using machine learning analysis. Neonatal dried blood spot (DBS) samples were collected at birth. (**B**) Distribution of SGA and appropriate for gestational age (AGA) as a function of GA and birth weight. (**C**) Measured lipids per class. Lipid classes included TGs, phosphatidylcholines (PCs), phosphatidylethanolamines (PEs), fatty acids (FAs), cholesteryl esters (CEs), diglycerides (DGs), lysophosphatidylcholines (LPs), sphingomyelins (SMs), and ceramides (CERs). (**D**) Principal components analysis of lipids colored by cohort.

The study enrolled 1390 pregnant women. Women with missing information regarding gestational age (GA) at delivery and with GA < 21 weeks at delivery were excluded, resulting in 1151 women across three cohorts: 533 from Kenya, 371 from Bangladesh, and 247 from Zimbabwe. Blood samples were collected between 6 and 24 weeks in Bangladesh, between 8 and 26 weeks in Zimbabwe, and between 20 and 24 weeks in Kenya (fig. S1). Analysis of plasma from these samples using targeted lipidomics and untargeted metabolomics produced 641 lipids and 639 metabolites (Fig. 1). Of 1151 pregnancies, 14.4% were preterm and 6.2% were SGA (third percentile, using Fenton charts) with Bangladesh having the highest prevalence of both PTB (25.6%) and SGA (7.2%), followed by Zimbabwe (PTB of 11.7% and SGA of 6.9%) and Kenya (PTB of 8.2% and SGA of 5.4%). The distribution of SGA versus appropriate for gestational age (AGA) as a function of GA and birthweight is shown in Fig. 1B, along with Fenton and Integrowth-21st growth charts. There were eight pregnancies with missing birth weight. These pregnancies were excluded from the SGA analysis. Only one pregnancy was both PTB and SGA (Fig. 1B). Cohort characteristics stratified by SGA and PTB outcomes are shown in Table 1. Overall cohort characteristics are shown in Table 2. Among collected demographic and clinical data, the only significant differences between SGA and AGA groups were observed for body mass index (BMI) (*P* = 0.03) and maternal age (*P* = 0.02) in the Kenyan data (Table 1) and for GA at sampling for PTB versus term groups across cohorts, but not within a cohort (Table 1).

**Table 1.** Summary statistics of SGA versus AGA infants and preterm versus term infants. BMI, body mass index.

		SGA vs. AGA				Preterm vs. term				
		AGA	SGA		*P* value	Term	Preterm		*P* value	
		1071 (93.7%)	72 (6.3%)			982 (85.3%)	169 (14.7%)			
Age		26.0 ± 5.6	26.7 ± 6.4		0.42	26.1 ± 5.6	25.7 ± 6.1		0.22	
BMI		25.3 ± 4.3	24.5 ± 4.2		0.12	25.2 ± 4.3	25.4 ± 4.2		0.51	
Gravida:	1	301 (28.1%)	18 (25.0%)		0.66	276 (28.1%)	45 (26.6%)		0.79	
	>1	770 (71.9%)	54 (75.0%)			706 (71.9%)	124 (73.4%)			
Para:	0	328 (30.6%)	20 (27.8%)		0.71	296 (30.1%)	55 (32.5%)		0.59	
	≥1	743 (69.4%)	52 (72.2%)			686 (69.9%)	114 (67.5%)			
GA at sampling		18.5 ± 3.6	18.0 ± 3.8		0.15	18.7 ± 3.6	14.5 ± 3.7		1 × 10^−5^	
Multiple births (*n*, %)		12 (1.1%)	3 (4.2%)		0.10	7 (0.7%)	8 (4.7%)		0.0001	
Infant sex, male (*n*, %)		549 (51.3%)	41 (56.9%)		0.42	505 (51.4%)	88 (52.1%)		0.94	
**Summary statistics of SGA and AGA infants: comparison among cohorts**										
		**Kenya**			**Bangladesh**			**Zimbabwe**		
		**AGA**	**SGA**	***P* value**	**AGA**	**SGA**	***P* value**	**AGA**	**SGA**	***P* value**
		504 (94.6%)	29 (5.4%)		337 (92.8%)	26 (7.2%)		230 (93.1%)	17 (6.9%)	
Age		27.1 ± 5.2	29.9 ± 6.4	0.02	23.8 ± 5.2	22.9 ± 5.0	0.44	26.7 ± 6.1	27.0 ± 5.6	0.78
BMI		24.9 ± 4.1	23.4 ± 4.1	0.03	26.3 ± 3.9	26.0 ± 3.6	0.81	24.8 ± 4.8	24.1 ± 4.5	0.51
Gravida:	1	74 (14.7%)	3 (10.3%)	0.78	152 (45.1%)	7 (26.9%)	0.11	75 (32.6%)	8 (47.0%)	0.34
	>1	430 (85.3%)	26 (89.7%)		185 (54.9%)	19 (73.1%)		155 (67.4%)	9 (53.0%)	
Para:	0	82 (16.3%)	3 (10.3%)	1	165 (49.0%)	9 (34.6%)	1	81 (35.2%)	8 (47.1%)	1
	≥1	422 (83.7%)	26 (89.7%)		172 (51.0%)	17 (65.4%)		149 (64.8%)	9 (52.9%)	
GA at sampling		21.3 ± 1.4	21.5 ± 1.6	0.58	15.6 ± 3.1	15.7 ± 3.0	0.91	16.9 ± 3.4	16.7 ± 3.1	0.66
Multiple birth, % (*n*)		0.02 (1)	3.4 (1)	0.11	0.09 (3)	0 (0)	1	6.5 (15)	11.7 (2)	0.31
Infant sex, male % (*n*)		53.1 (268)	51.8 (15)	1	51.3 (173)	57.7 (15)	0.55	47.0 (108)	64.7 (11)	0.21
HIV positive, % (*n*)		16.5 (83)	31.0 (9)	0.07				15.2 (35)	23.5 (4)	0.32
**Summary statistics of PTB and term infants: comparison among cohorts**										
		**Kenya**			**Bangladesh**			**Zimbabwe**		
		**Term**	**Preterm**	***P* value**	**Term**	**Preterm**	***P* value**	**Term**	**Preterm**	***P* value**
		489 (91.8%)	44 (8.2%)		275	96		218 (94.6%)	29 (5.4%)	
Age		27.3 ± 5.3	27.3 ± 6.2	0.83	23.5 ± 5.0	24.5 ± 5.7	0.21	26.6 ± 6.0	27.4 ± 6.3	0.47
BMI		24.8 ± 4.1	23.9 ± 4.2	0.04	26.3 ± 3.8	26.4 ± 4.0	0.81	24.7 ± 4.9	24.5 ± 4.2	0.81
Gravida:	1	69 (14.1%)	8 (18.2%)	0.61	130 (47.3%)	31 (32.3%)	0.01	77 (35.3%)	6 (20.7%)	0.009
	>1	420 (85.9%)	36 (81.8)		145 (52.7%)	65 (67.7%)		141 (64.7%)	23 (79.3%)	
Para:	0	76 (15.5%)	9 (20.5%)	0.52	140 (50.9%)	37 (38.5%)	0.05	80 (36.7%)	9 (31.0%)	0.7
	≥1	413 (84.5%)	35 (79.5%)		135 (49.1%)	59 (61.5%)		138 (63.3%)	20 (69.0%)	
GA at sampling		21.3 ± 1.5	21.4 ± 1.4	0.77	15.7 ± 2.9	15.9 ± 3.2	0.69	16.9 ± 3.4	16.7 ± 3.0	0.42
Multiple birth, % (*n*)		0.41% (2)	0% (0)	1	0.40% (1)	2.1% (2)	1	4.6% (10)	24.2% (7)	0.0004
Infant sex, male % (*n*)		54.0% (264)	43.2% (19)	0.22	49.4% (136)	57.3% (55)	0.22	48.2% (105)	48.3% (14)	1
HIV positive, % (*n*)		17.6% (86)	13.6% (6)	0.65				17.0% (37)	6.9% (2)	0.42

**Table 2.** Prevalence of outcomes and characteristics of mother-infant pairs in Kenya, Bangladesh, and Zimbabwe cohorts. BMI, body mass index.

		Kenya	Bangladesh	Zimbabwe	Total
Cohort		*n* = 533	*n* = 371	*n* = 247	*n* = 1151
GA at delivery		39.35 ± 2.1	37.52 ± 2.4	38.55 ± 2.2	38.55 ± 2.2
PTB (<37 weeks)		44 (8.2%)	96 (25.6%)	29 (11.7%)	169 (14.4%)
Early PTB (<32 weeks)		1 (0.2%)	11 (3.0%)	5 (2.0%)	17 (1.5%)
SGA (IG21)		32 (6.0%)	30 (8.2%)	20 (8.1%)	82 (7.1%)
SGA (Fenton)		29 (5.4%)	26 (7.2%)	17 (6.9%)	72 (6.2%)
PTB and SGA (Fenton)		0 (0%)	0 (0%)	1 (0.4%)	1 (0.08%)
Birth weight (grams)		3252 ± 449	2961 ± 462	2950 ± 510	3094 ± 489
Age at delivery		27.7 ± 7.8	23.7 ± 5.2	26.7 ± 6.1	26.2 ± 6.9
BMI		24.8 ± 4.1	26.3 ± 3.9	24.7 ± 4.8	25.3 ± 4.3
HIV positive		92 (17.2%)		39 (15.8%)	
Gravida:	1	77 (14.4%)	159 (43.8%)	83 (33.6%)	319 (27.9%)
	>1	456 (85.6%)	204 (56.2%)	164 (66.4%)	824 (72.1%)
Para:	0	85 (15.9%)	177 (47.7%)	89 (36.0%)	351 (30.5%)
	≥1	448 (84.1%)	194 (52.3%)	158 (64.0%)	800 (69.5%)
Multiple birth (*n*)		2	3	17	22
Infant sex (male)		283 (53.1%)	188 (51.8%)	119 (48.2%)	590 (51.2%)

### Maternal lipidome

#### Maternal lipidome in PTB pregnancies

**Fig. 2.**
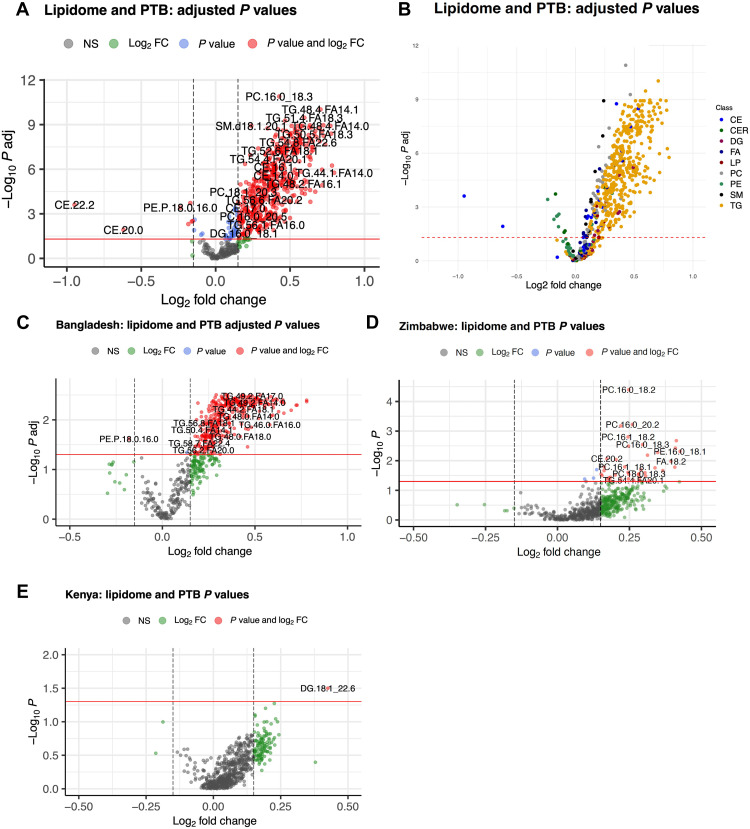
Volcano plots showing associations of maternal lipidome with PTB. (**A**) Overall associations in the full cohort colored by the significance of the association. A total of 526 lipids were significant (Wilcoxon rank sum test, *P* < 0.05) and 520 lipids were statistically significant after false discovery rate (FDR) adjustment (Wilcoxon rank sum test with Benjamini-Hochberg correction, adjusted *P* < 0.05). (**B**) Colored by lipid class. (**C**) Bangladesh. The strongest associations were observed in Bangladesh data where 415 lipids were significant (Wilcoxon rank sum test, *P* < 0.05) and 365 lipids were statistically significant after FDR adjustment (Wilcoxon rank sum test with Benjamini-Hochberg correction, adjusted *P* < 0.05). (**D**) Zimbabwe. There were 43 statistically significant changes (*P* < 0.05) and one statistically significant association after FDR adjustment [PC.16.0/18.2 with log(FC) = 0.25 and *P* = 0.026]. (**E**) Kenya. One lipid was significant (DG.18.1/22.6) (*P* < 0.05), with no statistically significant associations after FDR adjustment (adjusted *P* < 0.05). FC, fold change; NS, not significant.

In preterm compared to term pregnancies, differences in 526 lipids were significant (Wilcoxon rank sum test, *P* < 0.05), and 520 lipids were statistically significant after false discovery rate (FDR) adjustment (Wilcoxon rank sum test with Benjamini-Hochberg correction, adjusted *P* < 0.05), with almost all of these lipids having increased levels in preterm pregnancies {log transformed fold change [log(FC)] > 0} (Fig. 2A). The lipid class with the largest percentage of lipids showing statistically significant association with PTB was TG (*n* = 422, 81.1%) followed by phosphatidylcholines (PCs) (*n* = 31, 6%) (Fig. 2B). In addition, 20 cholesteryl esters (CEs), 14 FAs, 10 phosphatidylethanolamines (PEs), nine sphingomyelins (SMs), seven lysophosphatidylcholines (LPs), and seven diglycerides (DGs) had significant changes. Among TG class, the two most prevalent subclasses were TG containing omega-6 polyunsaturated FAs (PUFAs; 29.9%) and TGs containing saturated FAs (SAFAs; 24.4%). Among the FA, the most significant changes (increased levels) were observed in α-linolenic acid (FA18.3) [log(FC) = 0.4, adjusted *P* = 2.6 × 10^−6^], palmitoleic acid (FA16.1) [log(FC) = 0.34, adjusted *P* = 4.6 × 10^−6^], and myristoleic acid (FA14.1) (logFC = 0.2, adjusted *P* = 7.5 × 10^−6^). Once stratified by the cohort, we observed the strongest associations in Bangladesh data wherein 415 lipids were significant (*P* < 0.05) and 365 lipids were statistically significant after FDR adjustment (adjusted *P* < 0.05). In Zimbabwe data, 43 lipids were significant (*P* < 0.05) and one [PC16.0/18.2; *P* = 0.026, log(FC) = 0.25] remained significant after FDR adjustment (adjusted *P* < 0.05). In the Kenya data, one lipid was significant and none after FDR adjustment. The 30 lipids with the highest associations overall and for each country are listed in tables S2 to S5. Links to files containing the full lists of all significant lipids overall and for each country are available in Dryad (please see Data and materials availability).

To further analyze whether the time of sampling affected the associations, we stratified data in Bangladesh cohort, where the strongest associations among cohorts were observed, into two groups based on when sampling was performed: (i) ≤ 16 weeks’ GA and (ii) >16 weeks’ GA. We then analyzed associations in each period separately and observed the same qualitative performance for both time periods, confirming our results (fig. S2). In addition, we observed a stronger signal earlier in pregnancy, before 16 weeks’ GA.

#### Maternal lipidome in SGA pregnancies

**Fig. 3.**
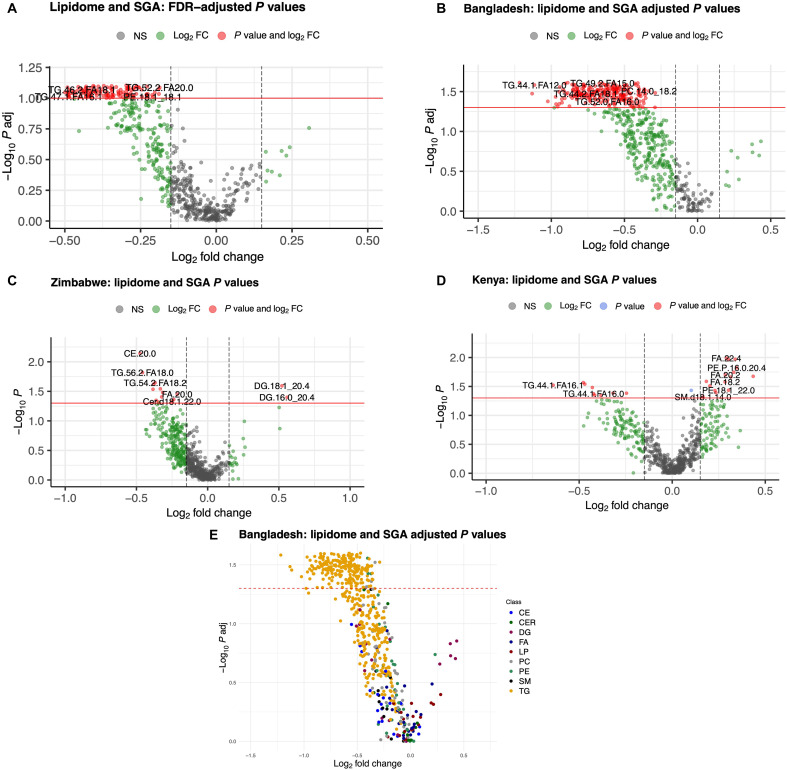
Volcano plots showing associations of lipidome with SGA across countries. (**A**) All cohorts. A total of 189 lipids were statistically significant (Wilcoxon rank sum test, *P* < 0.05) and 130 lipids were statistically significant after FDR adjustment (Wilcoxon rank sum test with Benjamini-Hochberg correction, adjusted *P* < 0.1). (**B**) Bangladesh. The strongest associations were observed in Bangladesh data where 325 lipids were significant (*P* < 0.05) and 242 lipids were statistically significant after FDR adjustment (adjusted *P* < 0.05). (**C** and **D**) Zimbabwe and Kenya, respectively. In the Zimbabwe and Kenya data, 15 and 25 lipids, respectively, were significant (*P* < 0.05), with no statistically significant associations after FDR adjustment (adjusted *P* < 0.05). (**E**) Associations in the Bangladesh data colored by class. FC, fold change; NS, not significant.

In pregnancies of SGA (as defined by Fenton growth curves) compared to term AGA infants across cohorts, 189 lipids were statistically significant (*P* < 0.05), and 130 lipids were statistically significant after FDR adjustment (adjusted *P* < 0.1), all of which were TGs (Fig. 3A). As in the case of PTB, the strongest changes in maternal lipids in SGA pregnancies were observed in Bangladesh data where 325 lipids had significant changes in SGA compared to those in term AGA pregnancies (Wilcoxon rank sum test, *P* < 0.05) and 242 lipids were statistically significant after FDR adjustment (Wilcoxon rank sum test with Benjamini-Hochberg correction, adjusted *P* < 0.05) (Fig. 3B). Opposite to PTB, all 242 lipids had decreased levels in SGA pregnancies. Among 242 lipids, 234 (96.7%) were TG (Fig. 3E). In addition, four PCs (PC14.0/18.2, PC14.0/20.4, PC18.0/18.3, and PC14.0/18.1), three PEs (PE16.0/18.2, PE16.0/18.1, and PE16.1/18.2), and a ceramide (CEM) (Cer.d18.1.24.0) had a significant decrease. Kenya and Zimbabwe had 25 and 15 significant associations (*P* < 0.05), respectively, and none stayed significant after FDR adjustment (adjusted *P* < 0.5) (Fig. 3, C and D). The 30 lipids with the highest associations overall and for each country are listed in tables S6 to S9. Links to files containing the full list of all significant lipids overall and for each country are available in Dryad (please see Data and materials availability).

We repeated the above analysis using the Intergrowth-21st classification for SGA. The observed changes in lipids were qualitatively similar to those seen with use of the Fenton classification of SGA in that most of lipids had lower levels in SGA pregnancies, but lower levels of significance in their associations were found in most cases. Specifically, 170 lipids had statistically significant changes in SGA compared with those in term AGA pregnancies (*P* < 0.05); all of the 170 lipids except one (DG14.1/18.2) showed lower levels in SGA pregnancies, but the significance did not hold after the FDR adjustment (fig. S3). As before, the largest number of changes occurred in Bangladesh with 244 statistically significant associations, whereas Kenya and Zimbabwe had 28 and 83 significant associations, respectively. None of the associations were significant after FDR adjustment.

#### Associations of lipid classes with PTB and SGA

**Fig. 4.**
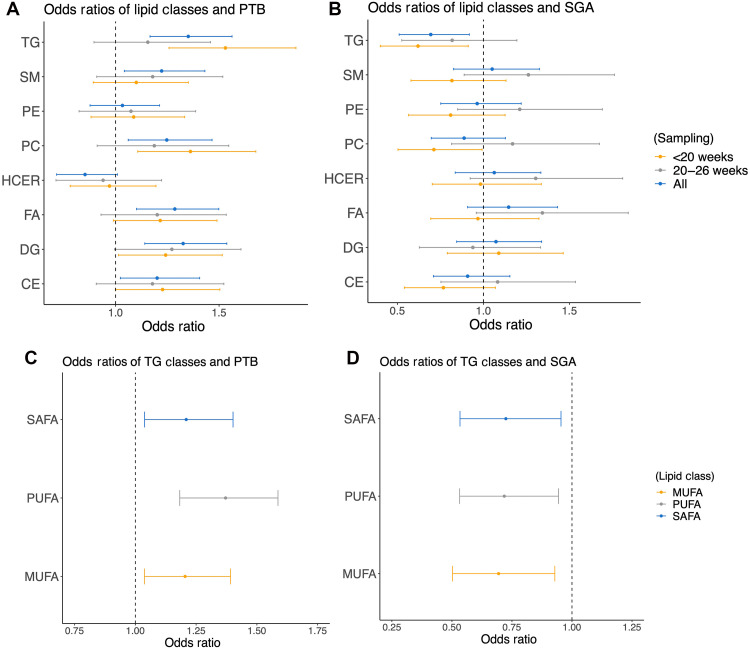
ORs for associations between lipid classes with PTB and SGA. (**A**) Odds ratios (ORs) for PTB stratified by time of sampling, showing the highest OR for TGs when sampling was performed before 20 weeks of pregnancy. (**B**) ORs for SGA stratified by time of sampling, showing the lowest OR for TGs sampled before 20 weeks of pregnancy. (**C**) ORs for saturated FAs (SAFA), monounsaturated FAs (MUFAs), and PUFAs in PTB, showing consistently elevated ORs. (**D**) ORs for SAFA, MUFA, and PUFA in SGA, showing consistently lower ORs.

To quantify the variations across cohorts, we analyzed regression coefficients between each lipid and PTB across cohorts. We observed that the values of the regression coefficients markedly differed across cohorts (fig. S4). To capture the overall impact on different lipid classes, we next investigated aggregated associations of classes of lipids with PTB and SGA. To address the issue of temporal changes of lipid levels, we stratified the data by time of sampling in terms of GA and separately analyzed samples obtained before 20 weeks of pregnancy, and samples obtained at 20 weeks or after. In line with results for univariate analysis shown in the volcano plots (Figs. 2 and 3), we observed the strongest association with TG, with increased odds ratios (ORs) for PTB (Fig. 4A and fig. S5) and decreased ORs for SGA (Fig. 4B) consistently in all three cases (<20 weeks’ GA, ≥20 weeks’ GA, and no stratification). For PTB, the highest OR was observed for TG when sampling was performed before 20 weeks of pregnancy [OR = 1.53 (1.26, 1.87)]. Analogously, for SGA, the lowest OR was observed for TG when sampling was performed before 20 weeks of pregnancy [OR = 0.62 (0.40, 0.91)]. We further calculated ORs separately for SAFAs, monounsaturated FAs (MUFAs), and PUFAs for PTB (Fig. 4C) and SGA (Fig. 4D), all showing elevated and decreased values for PTB and SGA, respectively.

### Maternal metabolome

#### Maternal metabolome in PTB pregnancies

**Fig. 5.**
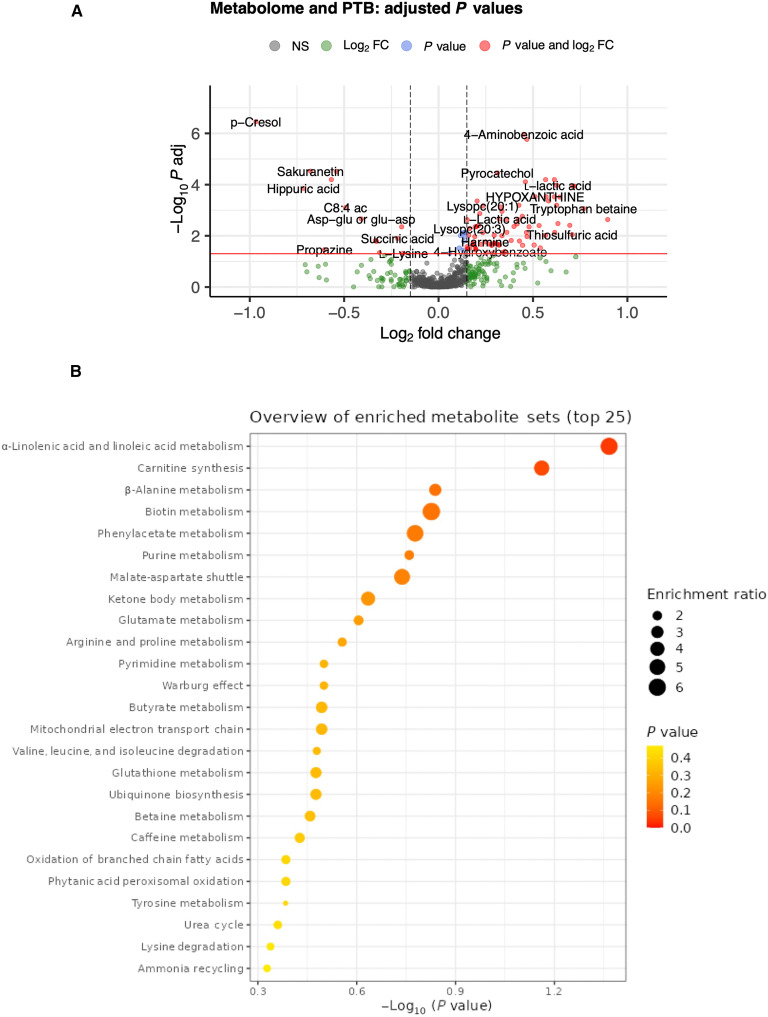
Metabolome and PTB. (**A**) Volcano plots showing association of metabolome with PTB. Colored by significance. A total of 154 lipids were significant (Wilcoxon rank sum test, *P* < 0.05) and 99 lipids were statistically significant after FDR adjustment (Wilcoxon rank sum test with Benjamini-Hochberg correction, adjusted *P* < 0.05). (**B**) Enriched metabolic pathways (*P* < 0.05). FC, fold change; NS, not significant.

Among the metabolic changes in preterm pregnancies, 154 metabolites were significant (Wilcoxon rank sum test, *P* < 0.05) and 99 metabolites were statistically significant after FDR adjustment (Wilcoxon rank sum test with Benjamini-Hochberg correction, adjusted *P* < 0.05) (Fig. 5A). We next performed pathway enrichment analysis on the basis of the list of significant metabolites. While not all compound identities could be matched to the MetaboAnalyst database (*11*), several enriched pathways were identified (Fig. 5B) with α-linolenic acid and linoleic acid metabolism having the highest statistical significance (*P* = 0.043) and highest enrichment, followed by carnitine synthesis metabolism (*P* = 0.069). However, significance did not hold for any enriched pathway after FDR adjustment, possibly because not all compound identities could be matched. Per country, 46, 66, and 14 metabolites show significant changes in Bangladesh, Zimbabwe, and Kenya, respectively, but none were significant after FDR adjustment (not shown).

#### Impact of the maternal to neonatal metabolome

**Fig. 6.**
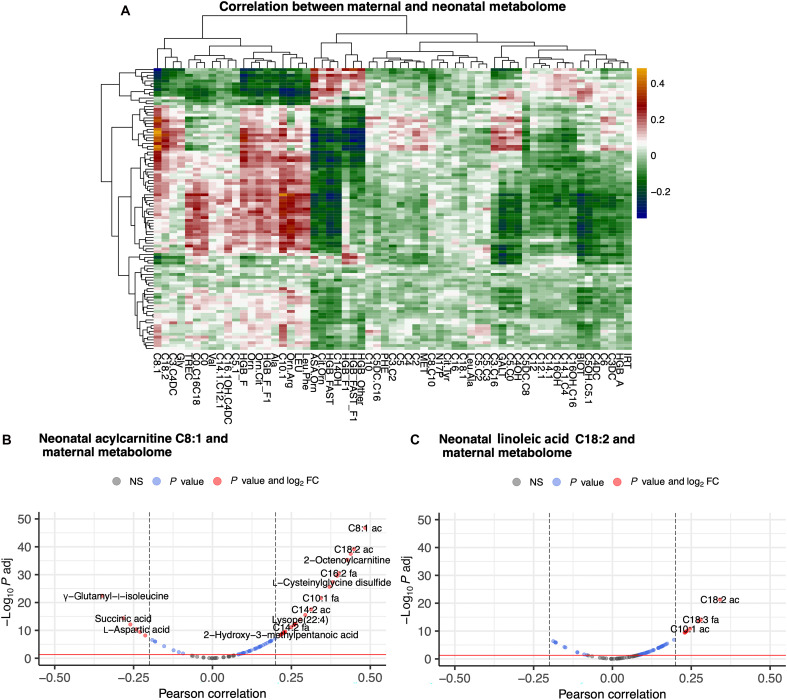
Correlation between maternal and neonatal metabolome. (**A**) Correlations between 99 maternal metabolites with 61 neonatal metabolites that were significantly changed in PTB. Among 6039 correlation values shown, 2409 (39.9%) were statistically significant (adjusted *P* < 0.05). The highest positive correlations can be observed for the neonatal metabolite acylcarnitine C8:1 with several maternal metabolites (indicated by yellow cells in the heatmap). Correlations with the maternal metabolome are also very high for Linoleoylcarnitine (C18:2). (**B**) Volcano plot showing associations between neonatal acylcarnitine C8:1 with maternal metabolites. The highest correlation was with maternal carnitine C8:1 (adjusted *P* < 10^−47^). (**C**) Volcano plot showing associations between neonatal Linoleoylcarnitine (C18:2) with maternal metabolites. The highest correlation was with the maternal linoleic acid (C18:2). FC, fold change; NS, not significant.

To investigate whether neonatal metabolomic changes at birth correlated with maternal metabolome, we computed Pearson correlation between 99 maternal metabolites with 61 neonatal metabolites that were significantly changed in PTB. Among 6039 correlation values obtained for each pair of a maternal and a neonatal metabolite, 2409 (39.9%) were statistically significant (adjusted *P* < 0.05). Correlation structure is shown in Fig. 6A.

We next investigated the top correlations, specifically neonatal acylcarnitine C8:1 and neonatal Linoleoylcarnitine (C18:2) with maternal metabolites. In these cases, the analysis shows direct linkages, that is, the correlations are the strongest between mom-baby metabolites of the same type. Specifically, the overall highest correlation of neonatal carnitine C8:1 is with maternal carnitine C8:1 (adjusted *P* < 10^−47^) (Fig. 6B). Neonatal C8:1 had high correlations with several other maternal carnitines and metabolites. Similarly, neonatal and maternal linoleic acid (C18:2) were strongly correlated (Fig. 6C).

#### Maternal metabolome in SGA pregnancies

**Fig. 7.**
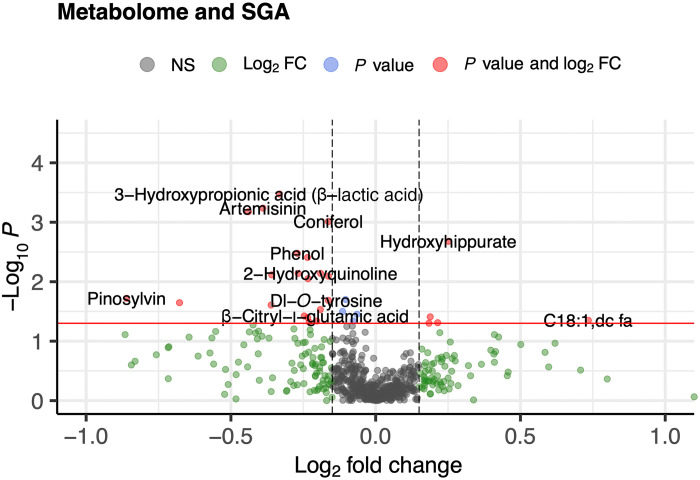
Volcano plots showing association of maternal metabolome with SGA. Thirty-one metabolites show significant changes, but none remain significant after FDR adjustment. FC, fold change; NS, not significant.

Analysis of associations between maternal metabolome and SGA based on Fenton classification showed 31 metabolites had significant changes, but none were significant after FDR adjustment (Fenton classification of SGA) (Fig. 7). Similarly, for SGA classified on the basis of Intergrowth-21st standards, 39 metabolites had significant changes, 74% of which had decreased levels in SGA, but none were significant after FDR adjustment (fig. S6).

### Machine learning prediction model and common biomarkers for PTB

**Fig. 8.**
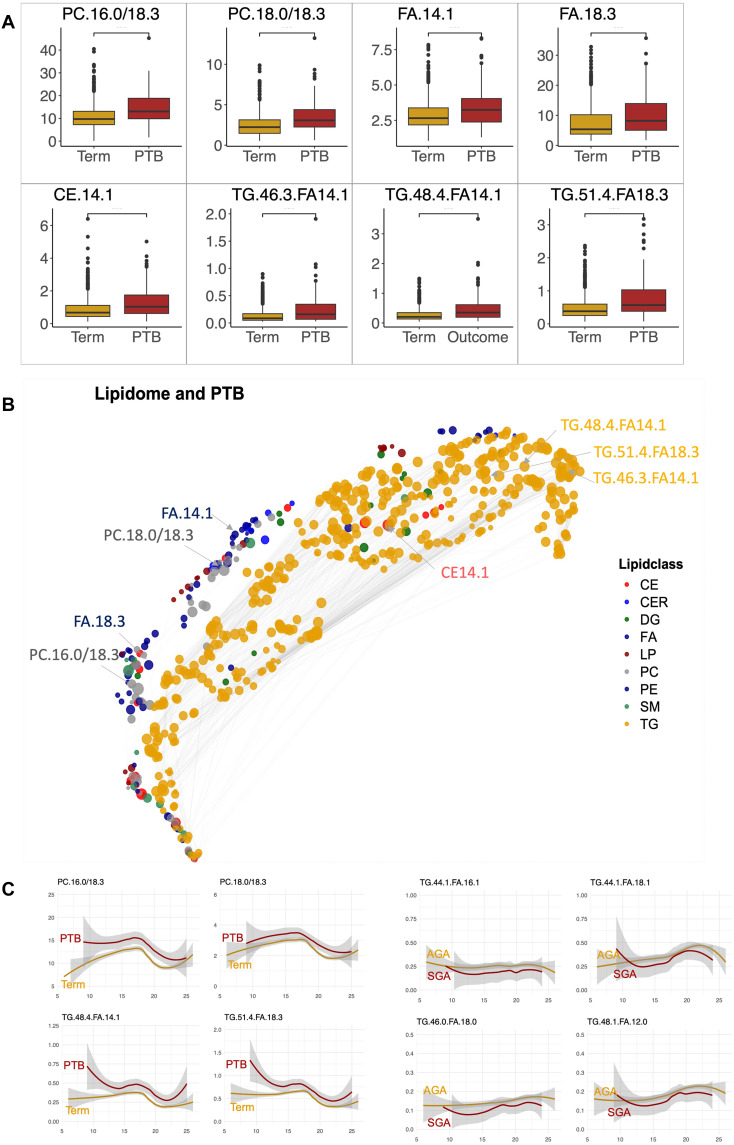
Biomarkers identified by the machine learning algorithm for early prediction of PTB from maternal lipidome. Observed prediction accuracy was AUC = 0.69, 95% CI = (0.68, 0.70). (**A**) Box plots of identified biomarkers comparing their values for PTB and term pregnancies. (**B**) Uniform manifold approximation and projection plot visualizing correlation structure of the lipidome and positions of lipid biomarkers selected by the machine learning model. (**C**) Top identified lipids for PTB and SGA as a function of time of sampling. Lipid classes: TGs, PCs, PEs, FAs, CEs, DGs, LPs, SMs, and CEMs.

We observed modest accuracy in predicting PTB from the lipidome, the metabolome, and demographic/clinical data [area under the receiver operator curve (AUC) = 0.69, 95% confidence interval (CI) = (0.68, 0.70)] using elastic net (EN). The performance of the lipidome and demographic/clinical data model was only slightly lower [AUC = 0.67, 95% CI = (0.66, 0.68)]. Prediction accuracy when using Stabl instead of EN had almost identical performance (table S9). The eight most predictive biomarkers identified by the lipidome model were three TGs (TG46.3.FA14.1, TG48.4.FA14.1, and TG51.4.FA18.3), two FAs [α-linolenic acid (FA18.3) and myristoleic acid (FA14.1)], one CE (CE14.1), and two PCs (PC16.0/18.3 and PC18.0/18.3) (Fig. 8A). All eight features were statistically significant (table S10), as determined by the univariate analysis. Correlation structure among all lipid features is shown in Fig. 8B, revealing clustering of lipids of the same class.

Eight biomarkers were identified using invariant causal prediction (ICP), revealing that they have consistent changes in PTB across the cohorts (fig. S7). Six of those biomarkers overlap with the biomarkers determined by Lasso (Fig. 8A): two FAs [α-linolenic acid (FA18.3), myristoleic acid (FA14.1)], a PC (PC16.0/18.3), and three TGs (TG46.3.FA14.1, TG48.4.FA14.1, and TG51.4.FA18.3). In addition, ICP identified two PEs (PE18.0/18.2 and PE16.0/18.2).

A machine learning model for predicting SGA birth had lower accuracy [AUC = 0.64, CI 95% = (0.62, 0.65)]. Prediction accuracy of all models is summarized in table S10. Visualized as a function of time of sampling, identified biomarkers show temporal variations but consistently increased or decreased levels in PTB and SGA, respectively (Fig. 8C).

## DISCUSSION

We found lipidomic and metabolomic changes early- to mid-gestation in pregnancies with a PTB or SGA outcome in cohorts of mothers from Bangladesh, Zimbabwe, and Kenya. While there were variations across cohorts, we consistently observed lipid levels that were increased in PTB and decreased in SGA relative to the term AGA pregnancies. Changes were observed most prominently in TGs, including increased TGs containing proinflammatory omega-6 PUFAs in PTB pregnancies. An aggregated analysis of classes of lipids stratified by time of sampling revealed that TGs increased with PTB and decreased with SGA. The greatest odds for increased or decreased TGs for PTB or SGA, respectively, were observed in samples obtained before 20 weeks of pregnancy, and the most significant changes were observed in the Bangladesh data. We observed significant correlations between maternal and neonatal metabolomes. Eight biomarkers predictive of PTB were identified by the machine learning model, including three TGs, two FAs (α-linolenic and myristoleic acids), a CE, and two PCs. Univariate analysis also identified each of these lipids as having statistically significant changes with PTB.

The predictive values of the machine learning algorithm from maternal lipidome/metabolome for PTB/SGA were modest. This is in contrast to a previous machine learning model developed on 17 metabolites that showed high accuracy (*12*). However, in that work, the 17 metabolites used in the model were preselected on the whole data, thus opening a possibility of overfitting and overoptimistic results. Instead, in our model, we used cross-validation to ensure that biomarkers were not seen by the test data. The modest performance of our machine learning algorithm is possibly due to relatively small values of fold change. While this predictive performance does not suffice for clinical utility, it does, however, demonstrate that there exist distinct maternal lipidomic/metabolic signatures of both PTB and SGA that precede these outcomes and point to several biomarkers. This also opens the possibility that enhancing/integrating these omics with other omics and/or clinical variables could lead to higher performance and thus better prediction.

Across cohorts, univariate analysis of PTB associations as well as ICP analysis revealed the most significant changes in PC16.0/18.3, which is functionally related to α-linolenic acid and hexadecanoic acid (*13*). In the Kenya data, the only lipid that had statistical significance was diacylglycerol that is structurally and functionally related to oleic acid. In the Zimbabwe data, the lipid with most significance was PC16:0/18:2, which is functionally related to linoleic acid (*14*).

Dyslipidemia in pregnancy has been previously associated with PTB in an epidemiological study (*15*), by measuring total levels of maternal blood lipids (e.g., total cholesterol and TGs) during pregnancy, and has been substantiated via a meta-analysis (*16*). Increased maternal lipid levels were associated with large-for-gestational age outcome in pregnancies complicated by gestational diabetes mellitus (*17*). More recently, omics analyses enabled closer examination of the metabolome and lipidome in association with PTB and SGA with mixed results. In a nested case control study of 100 women, metabolome analysis of 333 metabolites identified 38 markers, primarily FAs, associated with PTB (*12*). In follow-up work, lipidome analysis in the same cohort reported associations of seven lipids with spontaneous PTB and only one with SGA, possibly due to having only 16 cases of SGA in the cohort (*18*). Untargeted analysis of serum lipids identified lysophosphatidic acid (LPA20:4) as a biomarker of SGA (*19*). Analysis of the metabolome and lipidome stratified by BMI revealed dyslipidemia in both obese and underweight women (*20*). Associations between changes in the maternal plasma lipidome in the first and third trimesters with cord blood lipids and in some cases with birthweight have also been observed (*21*). Nuclear magnetic resonance–based metabolic fingerprinting from maternal plasma samples collected at delivery in 80 mothers showed that circulating lipids were lower in mothers with either SGA or intrauterine growth restricted offspring, with similar patterns of lipid profiles (*22*). In contrast, a study of 964 Dutch women found no associations between maternal serum metabolome measures with PTB or SGA (*23*).

This work has several strengths. First, it allowed profiling of pregnancy cohorts from three populations in LMICs where PTB and SGA birth outcomes have high prevalence. Furthermore, an accurate estimate of GA at delivery was obtained using ultrasound measurements in early or midpregnancy, and, thus, we had precise knowledge of the PTB outcome. Second, detailed omics profiling measured a large number of lipids and metabolites, enabling detailed association mapping in relation to PTB and SGA and revealing lipidomic imbalances in populations from LMICs. The large cohort size enabled of statistically significant associations on a lipid level. Last, samples obtained from both mothers and their neonates allowed for analyzing relationships between maternal and neonatal metabolomes.

A limitation of this study is that information to distinguish between spontaneous and medically indicated PTB was not available. Furthermore, we could not distinguish between subphenotypes of spontaneous PTB including placental abruption and preterm premature rupture of membranes.

Another limitation of our data is the relatively wide range of GAs at which maternal samples were collected and the differences in time of sampling across cohorts. This can pose a twofold challenge to the analysis: First, it can result in weaker overall associations as they may be affected by time-varying associations. Despite this challenge, we observed changes in lipid levels in both PTB and SGA pregnancies testifying to the robustness of these associations. Second, because all the samples from Kenya were obtained during 20 to 24 weeks of gestation and most of the samples from Bangladesh and Zimbabwe data were obtained before 20 weeks, there are variations both by country and the time of sampling. We addressed this limitation carefully in our analysis. First, we repeated the analysis separately per cohort, where timing variability is much less, thereby significantly reducing timing variability in each separate analysis. This analysis confirmed out results showing the same lipid imbalance in SGA and PTB as the overall findings. This is especially apparent for the Bangladesh cohort that had the highest prevalence of PTB. The same trends are observed in the other two cohorts, but the associations were not statistically significant, possibly due to smaller sample sizes and numbers of PTB cases. In addition, the OR analysis was also stratified by time of sampling, once again providing consistent results. The time of sampling was further accounted for in the machine learning analysis. Last, we stratified data in the Bangladesh cohort, where the strongest associations among cohorts were observed, into two groups on the basis of time of sampling and analyzed associations in each group separately. This further confirmed our results and revealed a stronger signal earlier in pregnancy, before 16 weeks’ GA. Together, all of the above results demonstrate robustness of these signatures as these significant changes in lipids levels persist in the overall cohort and per country and across a range of sampling times.

An additional limitation of this study is that data about diet of the enrolled pregnant women was not available. Dietary factors can modify lipid levels, and a diet rich in omega-3 FAs has been recommended for healthy pregnancy (*24*). Because the data about diet were not available, we could not assess whether measured lipid levels were affected by the diet. Instead, our data allowed us to identify direct associations between the lipids with PTB and SGA.

A final challenge to the computational analysis of lipidomics, in general, is the inability of the current technologies for lipidomic analyses to resolve structural isomers, lipids that have the same formula but a different structure. This, in turn, limits understanding of the specific pathways involved in the pathogenesis of a studied disease.

Among the identified TG with statistically significant associations with PTB, 29.9% were TGs containing proinflammatory omega-6 PUFAs [including arachidonic acid (AA), linoleic acid, and dihomo-γ-linolenic acid], including 25 TGs containing AA. Thus, these pregnancies are at a disposition of having increased proinflammatory omega-6 PUFAs and imbalance of omega-6 with omega-3 PUFAs. AA plays important roles in pregnancy and fetal development, and increased levels of AA can lead to preterm labor through its conversion into proinflammatory prostaglandins, which initiate uterine contractions and cervical ripening (*25*). These findings point to possible interventional strategies. An effective interventional strategy that is known to reduce AA’s conversion into proinflammatory prostaglandins (especially PGE_2_ and PGF_2_α) is by dietary supplementation with long-chain fatty acids such as Docosahexaenoic Acid (DHA) and Eicosapentaenoic Acid (EPA) that can mediate AA concentration and prevent production of proinflammatory prostaglandins (*24*). This treatment has been previously proposed (*24*), and our study provides further evidence in favor of this intervention.

In addition, there is evidence that increased levels maternal TGs are associated with endothelial dysfunction (*26*) that, in turn, can contribute to PTB (*27*).

This study describes a strong pattern of increased early and midpregnancy maternal lipids in PTB and decreased maternal lipids in SGA pregnancies, as well as strong correlations between the early and midpregnancy maternal metabolome with the neonatal metabolome at birth. The top correlations between maternal and neonatal metabolomes [observed for neonatal acylcarnitine C8:1 and Linoleoylcarnitine (C18:2)] showed direct links, the correlations being the strongest between mother-infant metabolites of the same type. These results indicate a direct influence of the maternal with the neonatal metabolome. The large size of our cohort enabled detailed examination and identification of statistically significant associations. These results confirm divergent and distinct pathways to PTB and SGA. While impact of the maternal metabolome measured late in pregnancy was shown to be strongly related to the neonatal metabolome (*28*), observed correlations between maternal and neonatal metabolomes in our work were observed early to midpregnancy, suggesting that this influence starts earlier.

A strength of this study is that it offers a detailed analysis of the maternal lipidome based on individual lipids, going beyond analysis per lipid class, and on a large cohort. This enabled the identification of specific lipids, including TGs with proinflammatory omega-6 PUFAs, associated with the outcomes. This, in turn, may offer more specific guidance for design of interventions, including specific diets and dietary supplements that could reduce the risks of PTB and SGA. As a first next step, the observed lipid changes in PTB and SGA need to be verified independently. Once confirmed, significantly changed lipids can potentially point to the affected pathways as well as the types of foods and/or supplements that contain these lipids, thereby providing guidance for specific interventions via dietary intake or supplements and informing randomized controlled trials that would confirm benefits of proposed interventions. Our analysis further indicates that these lipidomic changes occur early in pregnancy (before 16 weeks of gestation). If further confirmed in longitudinal studies, then identification of these early signatures would offer a longer window for intervention.

This study warrants further investigation of maternal lipids and their dynamic changes in pregnancy for prediction of PTB and SGA. In the data analyzed in this work, the greatest lipidomic changes occurred in the Bangladesh cohort, possibly due to earlier sampling times, thus suggesting stronger associations earlier in pregnancy. Future studies with longitudinal data sampled across the pregnancy timeline could test this hypothesis and quantify prediction accuracy at different times in pregnancy. Integration of proteome, shown previously to be predictive for other pregnancy outcomes (*29*), into the machine learning model may lead to more accurate prediction of these outcomes and identify the most predictive biomarkers.

Identifying biomarkers predictive of these outcomes could ultimately lead to developing diagnostic tests and possible treatments, both antenatal and postnatal, followed by protocols for their use in LMICs and globally. Once such diagnostic testing is in place, it may lead to closer pregnancy monitoring and significantly improve maternal and infant outcomes.

## MATERIALS AND METHODS

### Experimental design

#### Study design and sample collection

This was a study of three prospective cohorts of pregnant women and their neonates from three sites: Mirzapur, Bangladesh, led by the Child Health Research Foundation; Kisumu, Kenya, led by the Kenya Medical Research Institute; and Harare, Zimbabwe, led by the University of Zimbabwe. The cohorts from these sites were developed by a collaboration of the Stanford University School of Medicine with The Ottawa Hospital Research Institute. Pregnant women were enrolled at their first antenatal care visit, typically before 20 weeks of gestation. Informed written consent was obtained before study enrollment. Participants had an early pregnancy ultrasound for GA dating. At that time, whole-blood samples for plasma isolation were collected. Heel-prick dried blood spot (DBS) samples were collected at birth from neonates in these cohorts. Further details on the study design and sample collection can be found in (*30*, *31*).

### Definitions of outcomes

#### Small for gestational age

We classified SGA neonates as neonates whose birthweight is below third percentile for newborns of the same GA and sex. We used the two most commonly used international growth standards: (i) Fenton growth charts (*32*) and (ii) Integrowth-21st growth charts (*33**–**35*). This is consistent with World Health Organization recommendations for use of international standards for growth (*31*)*.* We used third percentile of the birth weight per GA to focus on the most affected infants that are the most likely to have fetal growth restriction. We then compared the two classifications on the basis of these two charts in our cohorts.

#### Preterm birth

PTB was defined as birth at less than 37 weeks’ GA.

### Lipidomic and metabolomic analyses

Lipidomics and metabolomics in maternal plasma were measured using targeted mass spectrometry (MS) and untargeted liquid chromatography–MS (LC-MS), respectively. These approaches have been previously applied in multiple studies on prediction of pregnancy outcomes (*15*, *29*, *36*). Metabolites and complex lipids were extracted by biphasic separation using cold methyl tert-butyl ether, methanol, and water in a deep-well plate format. Metabolite compounds were analyzed using a broad-spectrum untargeted LC-MS platform (*37*); complex lipids were obtained using a targeted MS-based procedure (*38*). Hydrophilic interaction liquid chromatography and reversed-phase liquid chromatography separation in positive and negative ionization modes were used to analyze metabolic extracts four times. Data from each mode were processed using Progenesis QI software (v2.3, Nonlinear Dynamics, Durham, NC). Metabolite annotation was performed using the metid package (*39*) from the tidyMass project (*40*). Metabolite abundances were described in spectral counts. Lipidyzer data were analyzed using the Lipidomics Workflow Manager (v1.0.5.0) software; each detected lipid concentration was calculated as an average intensity value of the analyte multiple reaction monitoring (MRM) relative to the average intensity of the most structurally similar internal standard MRM multiplied by its concentration. Lipid abundances were expressed as nanomoles per gram.

Newborn DBS underwent metabolic screening as part of the Newborn Screening Ontario program. The screening analyzed 94 blood metabolites and metabolite ratios from eight metabolic classes (table S1) (*31*).

### Statistical analysis

#### Univariate analysis

We first investigated univariate associations of the lipidome and metabolome with PTB and SGA. For each lipid and metabolite, *P* values were calculated using Wilcoxon rank sum test. Benjamini-Hochberg method was used to control the FDR for multiple hypothesis testing (*41*). Log(FC) was calculated to quantify the amount of change between the cases (i.e., PTB or SGA) and controls (term or AGA). Because maternal lipid levels change dynamically in pregnancy and may also vary across populations as they are affected by diet and lifestyle, results were calculated for the whole cohort and separately for each site and presented using volcano plots. We also analyzed these associations stratified by fetal sex. The metabolite set enrichment analysis using metabolites with statistically significant associations was performed using MetaboAnalyst (v6.0) (*11*).

Group sum of the abundance of individual lipids that belong to each lipid class were then calculated. Nine classes of lipids were measured: TG, PC, PE, FA, CE, DG, LP, SM, and CEM. For each class, regression analysis was run to calculate nonadjusted and adjusted ORs. Covariates used in adjustment were maternal age and BMI. Results were calculated for the whole cohort and after stratification by GA at sampling (<20 and ≥20 weeks). Correlations between maternal and neonatal metabolomes were assessed using Pearson correlation.

#### Machine learning analysis

Machine learning analyses used maternal lipidomics, metabolomics, and demographic/clinical data, including maternal age, BMI, infant sex, multiple birth data, and GA at sampling. Monte Carlo repeated cross-validation method was used to prevent overfitting. In each step, the samples were randomly split into a training set (80%) and a test set (20%), and a classification model for prediction was trained on the training set using EN regression method (*42*). EN has been used for prediction of pregnancy outcomes from omics data including prediction of preeclampsia (*29*) and GA (*28*). The outcome was then predicted for the test set using the trained EN model. These steps were repeated 100 times. In each iteration, the train and test sets were chosen at random. Performance was assessed using the AUC. In each step, an AUC was calculated for the test data, and the final AUC was obtained by averaging the AUCs from each run. The tuning of the two parameters of the EN algorithm (the shrinkage parameter λ that controls the amount of penalization and α parameter that linearly combines $L_{1}$ and $L_{2}$ penalizations) was performed using only the train data; we used R package glmnet for implementation of EN; and within the package, function glmnet was used to choose the shrinkage parameter λ by internal cross-validation on the training data. For comparison, we also trained and tested the recently proposed machine learning method, Stabl (*43*). The same cross-validation scheme was applied to evaluate the prediction accuracy of Stabl as in the case of the EN.

To determine biomarkers common across the three cohorts, we used ICP (*44*), an analytical approach that exploits data heterogeneity by analyzing predictions across different settings (in our case, three different cohorts) and identifies variables that show invariance in their predictive accuracy across settings. This is achieved by comparing the conditional distribution of the outcome given different subsets of variables and determining the ones that do not have a statistically significant change. ICP considers the found variables to be causal because their relationship with the outcome does not change when other variables or conditions change. R package InvariantCausalPrediction was used. Because it compares each subset of available variables, ICP can be computationally demanding. To reduce the number of computations, the ICP package allows for preselection of the variables by three different machine learning methods. We used preselection by Lasso, as a common sparsity-promoting regularization method. To visualize correlation structure of the lipidome, uniform manifold approximation and projection dimensionality reduction technique was used (*45*). R version 4.1.3 was used.

### Ethics

This study was approved by the Stanford University School of Medicine Institutional Review Board (44656), Ottawa Health Sciences Network Research Ethics Board (20180330-01H), the Kenya Medical Research Institute Scientific and Ethics Review Unit (SSC2880), the Bangladesh Institute of Child Health (BICH-ERC-01-01-2019), the Research Council of Zimbabwe (03755), and the Medical Research Council of Zimbabwe (MRCZ/A/2452).

Local research nurses and study coordinators obtained consent before enrollment in either the hospital or clinic setting at time of presentation to antenatal care or at time of delivery. Risks and benefits were discussed. Information was provided both verbally and in written explanation that participation was voluntary and that receipt of clinical care would not be influenced in any way by decision to enroll or not. In our research study sites, generally only mothers presented for clinical care. The second parent typically was not present or available at the time of enrollment. In cases where the second parent is present, risks and benefits of enrollment will be discussed with both parents. Readback or verbal confirmation of understanding regarding voluntariness of participation and the risks and benefits for study enrollment was obtained from the consenting party, depending on literacy level. Capacity to consent was determined by research nurses or study coordinators (for instance, by assessing level of distress.) If the patient changed their mind on enrollment, then they could discontinue participation at any time. Information regarding these processes was stated clearly in the consent document. The parent served as the legally authorized representative for a pediatric patient. The husband or legal partner was considered the legally authorized representative of the consenting mother. The principal investigators for this study were not present during consent to prevent enrollees from being influenced by their presence when deciding to participate.
