# Supplementary material for: Maternal lipidomic signatures of preterm and small-for-gestational-age newborn infants in low- and middle-income countries
Source: Sci Adv. 2025 Dec 3;11(49):eadu9145. doi: 10.1126/sciadv.adu9145 (PMC12674114; doi:10.1126/sciadv.adu9145)
Supplement: Supplementary file 1 — Tables S1 to S11 Figs. S1 to S7 [file sciadv.adu9145_sm.pdf]

Science Advances
AAAS

Supplementary Materials for

Maternal lipidomic signatures of preterm and small-for-gestational-age newborn infants in low- and middle-income countries

Ivana Marić *et al.*

Corresponding author: Ivana Marić, ivanam@stanford.edu

Sci. Adv. **11**, eadu9145 (2025)
DOI: 10.1126/sciadv.adu9145

This PDF file includes:

Tables S1 to S11
Figs. S1 to S7

**Table S1.** Neonatal metabolites and metabolite ratios measured in newborn screening.	
Acylcarnitines and Acylcarnitine Ratios	C0C2C3C16C18.Cit; C0; C0.C16C18; C10; C10.1; C12; C12.1; C14OH; C14; C14.1; C14.1.C12.1; C14.1.C16; C14.1.C4; C14.2; C16OH; C16OH.C16; C16; C16.1OH; C16.1OH.C4DC; C18OH; C18; C18.1OH; C18.1; C18.2; C2; C3DC; C3; C3.C0; C3.C16; C3.C2; C3.C4DC; C4DC; C4OH; C4; C5DC; C5DC.C16; C5DC.C5OH; C5DC.C8; C5OH; C5OH.C2; C5OH.C5.1; C5OH.C8; C5; C5.1; C5.C0; C5.C2; C5.C3; C6DC; C6; C8; C8.1; C8.C10; C8.C2
Amino Acids and Amino Acid Ratios	Alanine (Ala); Arginine (Arg); Citrulline (Cit); Glycine (Gly); Leucine (Leu); Methionine (Met); Ornithine (Orn); Phenylalanine (Phe); Tyrosine (Tyr); Valine (Val) Cit:Arg; Cit:Orn; Cit:Tyr; Leu:Ala; Leu:Phe; Met:Phe; Orn:Arg; Orn:Cit; Orn:Phe; Phe:Tyr; Tyr:Phe; Val:Ala; Val:Phe;
Cystic Fibrosis Markers	Immunoreactive trypsinogen (IRT)
Endocrine Markers	Thyroid Stimulating Hormone (TSH)
Enzyme Markers	Biotinidase (BIOT); Galactose-1-phosphate-uridylyltransferase (GALT)
Hemoglobin	HGB_A; HGB_F1; HGB_FAST; HGB_FAST_F1; HGB_F; HGB_F_F1; HGB_Other
T-cell Function	T-Cell Receptor Excision Circles (TREC)
Others	Arginosuccinic acid/acidosis (ASA); ASA:Arg; ASA:Orn; MCA; N17P; SUAC

Table S2. Top 30 significant lipids for PTB (adjusted p-value<0.05)

SignificantLipidsPTB

Name	pvalue	BHpvalue	logFCvalue	meancases	meancontrols
PC.16.0_18.3	2E-14	1.277E-11	0.4272	14.7259	10.8688
TG.48.4.FA14.1	3E-13	9.526E-11	0.7044	0.4798	0.2758
TG.51.4.FA18.3	1.62E-12	3.4651E-10	0.5961	0.7547	0.4767
PC.18.0_18.3	3.42E-12	5.4747E-10	0.4666	3.4524	2.4908
TG.50.5.FA16.1	9.63E-12	1.10341E-09	0.7234	0.6735	0.388
TG.54.7.FA20.4	1.033E-11	1.10341E-09	0.6332	3.0584	2.1324
TG.54.6.FA20.3	1.421E-11	1.21174E-09	0.552	1.3747	0.9844
TG.54.6.FA16.1	1.533E-11	1.21174E-09	0.5706	1.8301	1.2525
TG.48.4.FA14.0	1.796E-11	1.21174E-09	0.7712	0.4409	0.2489
TG.52.6.FA16.0	1.954E-11	1.21174E-09	0.5842	1.5384	1.06
SM.d18.1.20.1	2.115E-11	1.21174E-09	0.2398	10.2611	8.7052
TG.54.5.FA16.1	2.268E-11	1.21174E-09	0.5107	1.854	1.3109
TG.52.6.FA20.4	3.295E-11	1.55285E-09	0.6417	1.5434	1.0229
TG.48.4.FA16.0	3.392E-11	1.55285E-09	0.6286	0.5464	0.3293
CE.18.3	4.27E-11	1.78118E-09	0.3487	64.6327	51.2946
TG.52.6.FA16.1	4.446E-11	1.78118E-09	0.6977	2.3524	1.586
TG.51.4.FA16.1	5.589E-11	2.04037E-09	0.5325	0.4765	0.3201
TG.50.5.FA18.1	5.908E-11	2.04037E-09	0.4188	0.3001	0.2197
TG.54.7.FA16.1	6.048E-11	2.04037E-09	0.5679	1.2333	0.8508
TG.52.4.FA18.0	8.021E-11	2.54806E-09	0.4512	0.5947	0.4354
TG.56.7.FA22.4	8.348E-11	2.54806E-09	0.5421	0.4999	0.3506
TG.53.4.FA18.3	9.208E-11	2.68285E-09	0.4752	0.5416	0.3812
TG.54.8.FA20.4	9.807E-11	2.7332E-09	0.6695	0.3599	0.243
TG.48.4.FA16.1	1.0654E-10	2.84549E-09	0.7143	0.5946	0.3352
CE.14.1	1.2925E-10	3.31388E-09	0.5346	1.3086	0.8879
TG.52.5.FA18.1	1.4864E-10	3.66465E-09	0.5107	5.5803	4.0046
TG.48.4.FA18.3	1.7144E-10	4.07006E-09	0.7765	0.7383	0.387
TG.51.3.FA17.0	1.9349E-10	4.41859E-09	0.4997	0.8708	0.59
TG.50.5.FA18.3	1.9991E-10	4.41859E-09	0.6628	1.2903	0.8395
TG.52.5.FA20.3	2.2008E-10	4.56551E-09	0.5664	0.7605	0.5201

Table S3. Top 30 significant lipids for PTB: Bangladesh (adjusted p-value<0.05)

SignificantLipidsPTB_Bangladesh

Name	pvalue	BHpvalue	logFCvalue
TG.49.2.FA17.0	3.893254828E-05	0.00413431328208	0.4849
TG.46.3.FA14.1	5.082015938E-05	0.00413431328208	0.5681
TG.48.4.FA14.0	5.106085365E-05	0.00413431328208	0.5099
TG.46.2.FA14.0	6.487409673E-05	0.00413431328208	0.5862
TG.55.5.FA20.4	6.86035694E-05	0.00413431328208	0.3563
TG.49.2.FA14.0	9.301652972E-05	0.00413431328208	0.4984
TG.50.2.FA14.1	0.00012661328335	0.00413431328208	0.3857
TG.49.3.FA15.0	0.00015970356578	0.00413431328208	0.4097
TG.48.4.FA18.3	0.00016328208029	0.00413431328208	0.5009
TG.53.5.FA20.4	0.00016619907026	0.00413431328208	0.4139
TG.50.3.FA18.0	0.0001721807327	0.00413431328208	0.3504
TG.47.2.FA15.0	0.00018073671791	0.00413431328208	0.5333
TG.46.2.FA12.0	0.00018153404469	0.00413431328208	0.5292
TG.51.2.FA16.1	0.00018233467761	0.00413431328208	0.4357
TG.52.6.FA20.4	0.00018638788123	0.00413431328208	0.4012
TG.46.3.FA12.0	0.00018886029844	0.00413431328208	0.5247
TG.47.2.FA14.0	0.00018886029844	0.00413431328208	0.5315
TG.44.1.FA14.0	0.00018969126466	0.00413431328208	0.7791
TG.48.4.FA14.1	0.00019474966885	0.00413431328208	0.4346
TG.52.6.FA14.0	0.00019560496143	0.00413431328208	0.4411
TG.47.1.FA15.0	0.00019906155359	0.00413431328208	0.614
TG.46.3.FA18.2	0.0002070498354	0.00413431328208	0.4173
TG.48.4.FA16.1	0.00021161689726	0.00413431328208	0.4479
TG.50.2.FA18.0	0.00022199954737	0.00413431328208	0.3611
TG.46.3.FA18.1	0.00022393794513	0.00413431328208	0.3503
TG.55.3.FA18.1	0.00023084817931	0.00413431328208	0.3567
TG.52.5.FA14.0	0.00023899047898	0.00413431328208	0.3961
TG.46.3.FA14.0	0.00024633526835	0.00413431328208	0.5017
PC.16.0_18.3	0.00024954740414	0.00413431328208	0.2752
TG.52.7.FA18.1	0.00025608987916	0.00413431328208	0.3533

Table S4. Significant lipids for PTB: Kenya (p<0.1)

SignificantLipidsPTB_Kenya

Name	pvalue	BHpvalue	logFCvalue
DG.18.1_22.6	0.03157742639355	0.99386682456553	0.4253
TG.58.5.FA18.1	0.0533546816365	0.99386682456553	0.2264
TG.60.10.FA22.5	0.06295926795877	0.99386682456553	0.1937
TG.56.4.FA20.3	0.07921863428532	0.99386682456553	0.1546
CE.16.1	0.0822347266637	0.99386682456553	0.1545
TG.58.6.FA22.5	0.09066970657393	0.99386682456553	0.2314
TG.58.6.FA16.0	0.09978707204689	0.99386682456553	0.1985
TG.54.6.FA22.5	0.09999769464107	0.99386682456553	0.2383

Table S5. Top 30 significant lipids for PTB: Zimbabwe (p-value<0.05)

SignificantLipidsPTB_Zimbabwe

Name	pvalue	BHpvalue	logFCvalue
PC.16.0_18.2	4.009398105E-05	0.02570024185369	0.2477
PC.16.0_20.2	0.00059936805661	0.14320992265433	0.2602
CE.18.2	0.00067024924799	0.14320992265433	0.2196
PC.16.1_18.2	0.00157347989479	0.25215015313972	0.2452
PE.16.0_18.2	0.00210491411731	0.2698499898392	0.4128
PC.16.0_18.3	0.00284767446686	0.29574554545942	0.2957
PC.16.0_18.1	0.00322967054324	0.29574554545942	0.2466
PE.16.0_20.4	0.00375602264308	0.30095131427696	0.4095
PE.16.0_18.1	0.00483649106445	0.33779431535753	0.4258
PE.18.1_20.4	0.00526980211166	0.33779431535753	0.3482
PC.18.1_20.2	0.0065104864597	0.37938380187869	0.3127
CE.20.2	0.00854849096881	0.44975611684553	0.1715
FA.14.1	0.00912141890638	0.44975611684553	0.2033
FA.18.2	0.01053755227205	0.48246935759901	0.3944
PC.16.1_18.1	0.01614983592354	0.62013497973614	0.2337
SM.d18.1.20.1	0.01614983592354	0.62013497973614	0.1623
FA.18.3	0.01652036061817	0.62013497973614	0.4075
TG.54.2.FA20.0	0.01741408679446	0.62013497973614	0.3387
PC.18.0_18.2	0.02005689451462	0.62886855977752	0.1362
FA.18.1	0.02035447289079	0.62886855977752	0.2898
CE.16.0	0.02158363231686	0.62886855977752	0.1618
PC.16.0_16.1	0.02158363231686	0.62886855977752	0.3662
PC.18.2_20.0	0.02476479378138	0.66730293320325	0.2451
FA.20.2	0.02548400556018	0.66730293320325	0.3038
PC.18.0_18.3	0.02755573781779	0.66730293320325	0.283
PC.16.0_20.4	0.02834431465586	0.66730293320325	0.1513
PC.16.0_20.1	0.02894843623168	0.66730293320325	0.2478
FA.16.1	0.02935731377198	0.66730293320325	0.2811
Cer.d18.1.24.0	0.03018999229781	0.66730293320325	0.2006
CE.18.1	0.03169605198342	0.67377881327152	0.1547

Table S6. Top 30 significant lipids for SGA (adjusted p-value<0.1)

SignificantLipidsSGA

Name	pvalue	BHpvalue	logFCvalue
TG.44.1.FA16.1	0.000685	0.088704	-0.6947
TG.44.1.FA18.1	0.000925	0.088704	-0.5308
TG.46.2.FA18.1	0.000932	0.088704	-0.4429
TG.48.1.FA12.0	0.000957	0.088704	-0.4913
TG.46.0.FA18.0	0.001081	0.088704	-0.5883
TG.44.2.FA16.0	0.001098	0.088704	-0.4898
TG.46.3.FA18.1	0.001308	0.088704	-0.3236
TG.44.2.FA18.2	0.001655	0.088704	-0.4645
TG.47.1.FA16.1	0.001798	0.088704	-0.4872
TG.47.2.FA14.0	0.002399	0.088704	-0.4366
TG.48.3.FA18.1	0.002648	0.088704	-0.2937
TG.46.0.FA12.0	0.002739	0.088704	-0.5134
TG.44.1.FA16.0	0.002823	0.088704	-0.5388
TG.46.3.FA18.2	0.002868	0.088704	-0.3592
TG.50.2.FA18.0	0.002988	0.088704	-0.2908
TG.46.1.FA18.1	0.003075	0.088704	-0.4785
TG.47.2.FA18.2	0.003245	0.088704	-0.3831
TG.46.3.FA12.0	0.003343	0.088704	-0.4459
TG.46.2.FA12.0	0.003455	0.088704	-0.4308
TG.48.2.FA18.1	0.003501	0.088704	-0.4308
TG.46.2.FA16.0	0.003522	0.088704	-0.4291
TG.48.1.FA18.0	0.003606	0.088704	-0.4374
TG.45.0.FA16.0	0.003653	0.088704	-0.5064
TG.46.2.FA18.2	0.003888	0.088704	-0.4252
TG.49.2.FA15.0	0.003989	0.088704	-0.3148
TG.46.1.FA12.0	0.004348	0.088704	-0.4851
TG.44.1.FA14.0	0.004384	0.088704	-0.6473
TG.47.0.FA14.0	0.004769	0.088704	-0.4695
TG.48.2.FA12.0	0.004797	0.088704	-0.3568
TG.47.2.FA15.0	0.004819	0.088704	-0.4126

Table S7. Top 30 significant lipids for SGA in Bangladesh (adjusted p-value<0.05)

BangladeshSignificantLipidsSGA

Name	pvalue	BHpvalue	logFCvalue
TG.46.3.FA18.2	0.000259	0.030016	-0.7297
TG.48.3.FA18.1	0.000533	0.030016	-0.6122
TG.50.2.FA14.1	0.000549	0.030016	-0.5476
TG.44.2.FA18.1	0.000561	0.030016	-0.7643
TG.51.2.FA17.0	0.000586	0.030016	-0.7196
TG.50.3.FA18.1	0.000598	0.030016	-0.6798
TG.48.3.FA18.2	0.00069	0.030016	-0.6308
TG.46.2.FA18.1	0.000828	0.030016	-0.7904
TG.47.2.FA18.2	0.000876	0.030016	-0.764
TG.50.3.FA18.2	0.000926	0.030016	-0.7012
TG.49.2.FA15.0	0.001013	0.030016	-0.6506
TG.49.3.FA18.2	0.001085	0.030016	-0.5309
TG.51.2.FA18.2	0.0011	0.030016	-0.5105
TG.49.2.FA18.2	0.001244	0.030016	-0.5257
TG.48.3.FA14.1	0.001322	0.030016	-0.6831
TG.50.2.FA18.0	0.001367	0.030016	-0.5416
TG.52.8.FA18.2	0.001404	0.030016	-0.4771
TG.49.2.FA14.0	0.001433	0.030016	-0.6713
TG.53.1.FA18.0	0.001443	0.030016	-0.5537
TG.50.3.FA18.0	0.001482	0.030016	-0.5075
TG.46.3.FA18.1	0.001492	0.030016	-0.4967
PC.14.0_18.2	0.001502	0.030016	-0.3331
TG.49.3.FA15.0	0.001605	0.030016	-0.6055
TG.47.2.FA15.0	0.001659	0.030016	-0.8441
TG.50.3.FA14.1	0.001682	0.030016	-0.4993
TG.46.3.FA16.0	0.001715	0.030016	-0.7407
TG.51.4.FA20.4	0.001738	0.030016	-0.7902
TG.49.1.FA14.0	0.00175	0.030016	-0.7912
TG.44.2.FA16.0	0.001761	0.030016	-0.7789

Table S8. Significant lipids for SGA in Kenya (p-value<0.05)

KenyaSignificantLipidsSGA

Name	pvalue	BHpvalue	logFCvalue
FA.22.4	0.010285	0.727279	0.291
PE.P.18.1.20.4	0.010735	0.727279	0.3018
FA.22.6	0.010735	0.727279	0.3384
PE.P.16.0.20.4	0.01506	0.727279	0.3461
FA.20.4	0.017959	0.727279	0.3307
FA.20.2	0.019977	0.727279	0.2854
TG.58.10.FA20.5	0.021128	0.727279	0.4349
PE.P.18.0.20.4	0.021689	0.727279	0.3149
PE.18.0_18.0	0.025826	0.727279	0.1835
FA.18.2	0.026409	0.727279	0.2838
TG.44.2.FA14.0	0.02709	0.727279	-0.4778
TG.44.2.FA18.2	0.028767	0.727279	-0.4696
TG.44.1.FA16.1	0.03006	0.727279	-0.6397
FA.22.2	0.030917	0.727279	0.2023
TG.47.2.FA16.1	0.032998	0.727279	-0.4291
PE.18.1_22.0	0.036734	0.727279	0.3026
FA.14.0	0.036958	0.727279	0.1022
TG.58.6.FA20.4	0.037071	0.727279	0.2295
TG.58.9.FA22.5	0.04046	0.727279	0.2346
TG.46.2.FA18.1	0.041193	0.727279	-0.318
TG.50.4.FA18.1	0.041316	0.727279	-0.2455
TG.44.1.FA16.0	0.042188	0.727279	-0.4229
TG.47.2.FA14.0	0.044764	0.727279	-0.4165
TG.44.1.FA18.1	0.045162	0.727279	-0.3828
SM.d18.1.14.0	0.048029	0.727279	0.1657

Table S9. Significant lipids for SGA in Zimbabwe (p-value<0.05)

ZimbabweSignificantLipidsSGA

Name	pvalue	BHpvalue	logFCvalue
CE.20.0	0.007196	0.956602	-0.4752
TG.56.2.FA18.0	0.015211	0.956602	-0.4574
TG.54.2.FA18.2	0.022634	0.956602	-0.3689
TG.56.3.FA20.2	0.023917	0.956602	-0.3797
DG.18.1_20.4	0.025494	0.956602	0.5214
TG.54.3.FA18.0	0.028662	0.956602	-0.3322
TG.54.3.FA18.3	0.029179	0.956602	-0.3838
TG.54.3.FA18.2	0.033022	0.956602	-0.3159
FA.20.0	0.035407	0.956602	-0.2133
TG.54.2.FA18.0	0.039259	0.956602	-0.3227
DG.16.0_20.4	0.039935	0.956602	0.5579
PC.14.0_18.2	0.043462	0.956602	-0.2382
TG.54.4.FA18.3	0.044945	0.956602	-0.3615
Cer.d18.1.22.0	0.046858	0.956602	-0.2496
TG.54.1.FA18.0	0.048038	0.956602	-0.3444

**Table S10.** P-values of lipid biomarkers determined by the ML algorithm.			
Biomarker	p-value	Adjusted p-value	LogFC
TG46.3.FA14.1	1.06e-09	1.3e-08	0.70
TG48.4.FA14.1	2.9e-13	6.3e-11	0.70
TG50.5.FA16.1	9.6e-12	9.4e-10	0.72
TG51.4.FA18.3	1.6e-12	2.6e-10	0.6
α-linolenic acid (FA18.3)	7.5e-07	2.6e-06	0.4
myristoleic acid (FA14.1)	2.6e-06	7.5e-06	0.2
CE14.1	1.3e-10	3.2e-09	0.5
PC16.0/18.3	2.0e-14	6.4e-12	0.4

**Table S11.** Predictive accuracy of machine learning models. Elastic net (EN) and Stabl were evaluated with and without penalization of demographic variables.				
	EN with penalized demographics		STABL with penalized demographics	
	PTB	SGA	PTB	SGA
Lipidome	**0.67 (0.66, 0.68)**	0.60 (0.6,0.62)	0.70 (0.69,0.71)	0.61 (0.60,0.62)
Metabolome	0.64 (0.63,0.65)	0.56 (0.55, 0.57)	0.63 (0.61,0.64)	0.55 (0.54,0.56)
Lipidome & Metabolome	**0.69 (0.68,0.699)**	0.57 (0.56,0.58)	0.69 (0.67,0.70)	0.57 (0.55 , 0.58)
	EN with unpenalized demographics		STABL with unpenalized demographics	
	PTB	SGA	PTB	SGA
Lipidome	**0.67 (0.66, 0.68)**	**0.64 (0.62,0.65)**	0.68 (0.69,0.71)	0.63 (0.60,0.62)
Metabolome	0.61 (0.6,0.63)	0.63 (0.62,0.64)	0.60 (0.61,0.64)	0.60 (0.54,0.56)
Lipidome & Metabolome	**0.69 (0.68,0.699)**	0.62 (0.6,0.63)	0.67 (0.67,0.70)	0.61 (0.55 ,0.58)

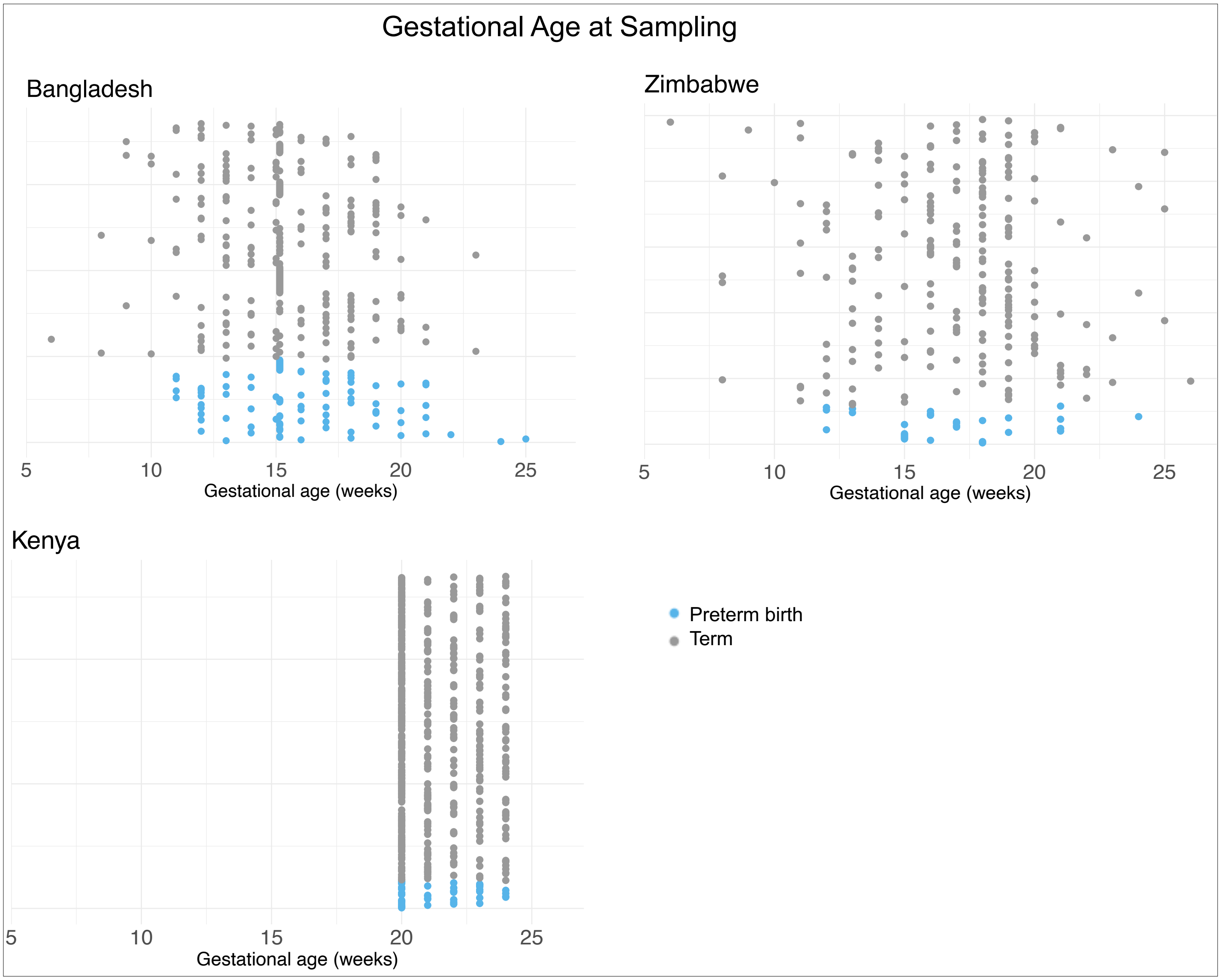

Figure S1. Gestational age at sampling for each of the three cohorts. Samples were collected between 6-24 weeks in Bangladesh, 8-26 in Zimbabwe and 20-24 weeks in Kenya.

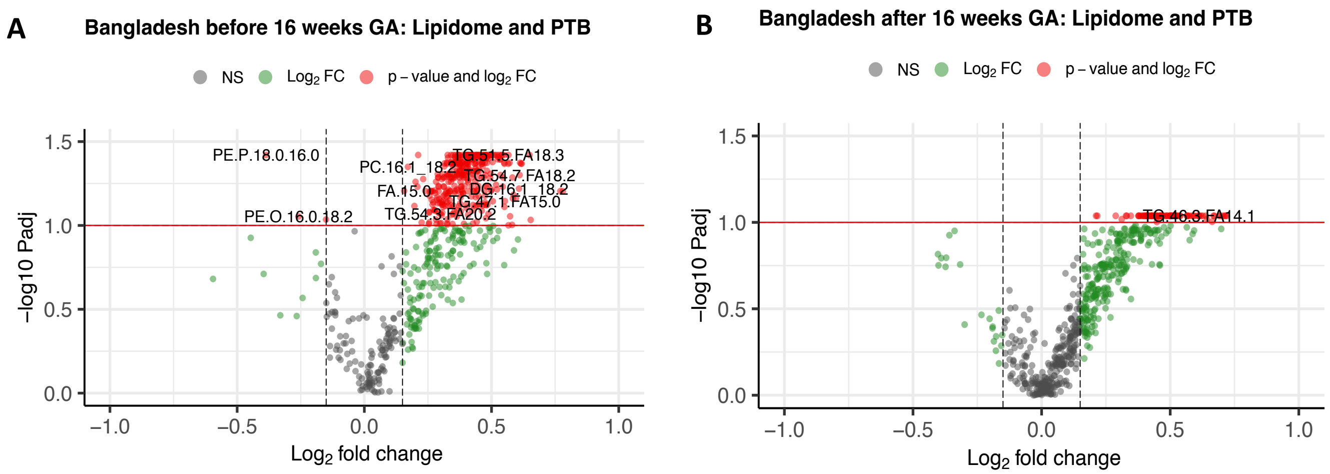

Figure S2. Volcano plots showing associations of maternal lipidome with preterm birth (PTB) in Bangladesh cohort and stratified by time of sampling. A. Before 16 weeks of gestational age **B.** After 16 weeks gestational age.

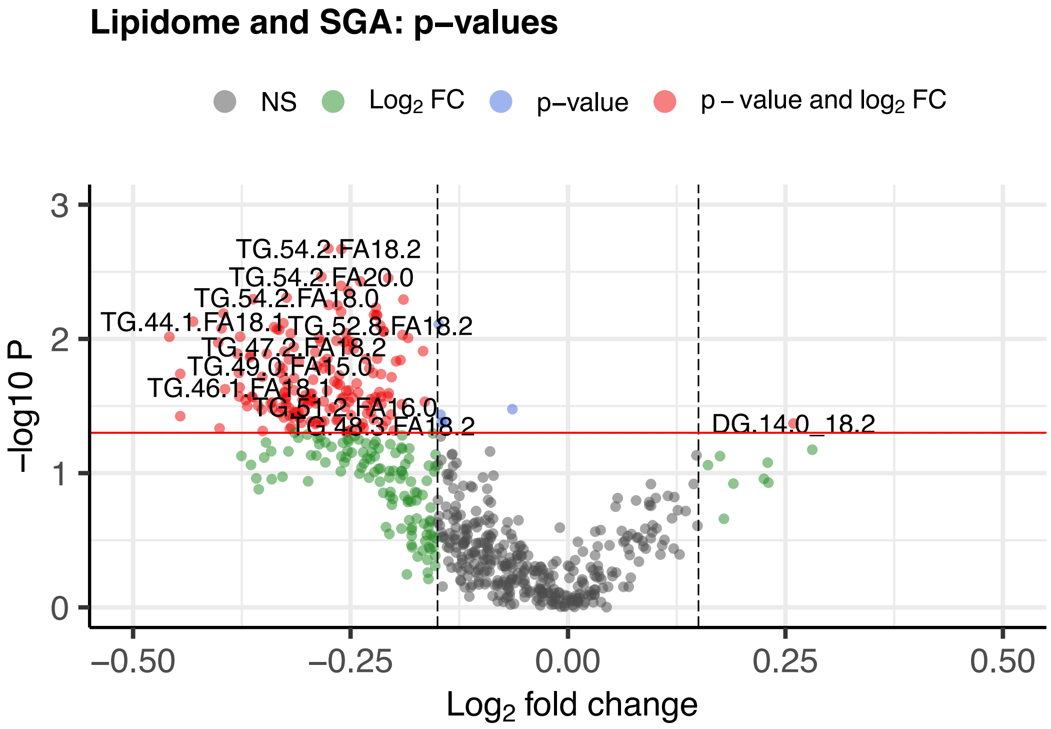

Figure S3. Volcano plots showing association of lipidome with SGA using Intergrowth-21 Classification. 170 lipids had statistically significant changes (p-value<0.05) but none were statistically significant after FDR-adjustment (adjusted p-value < 0.5). Among the 170 lipids, only one had increased levels in SGA (DG14.1/18.2) and all the rest had decreased levels.

A

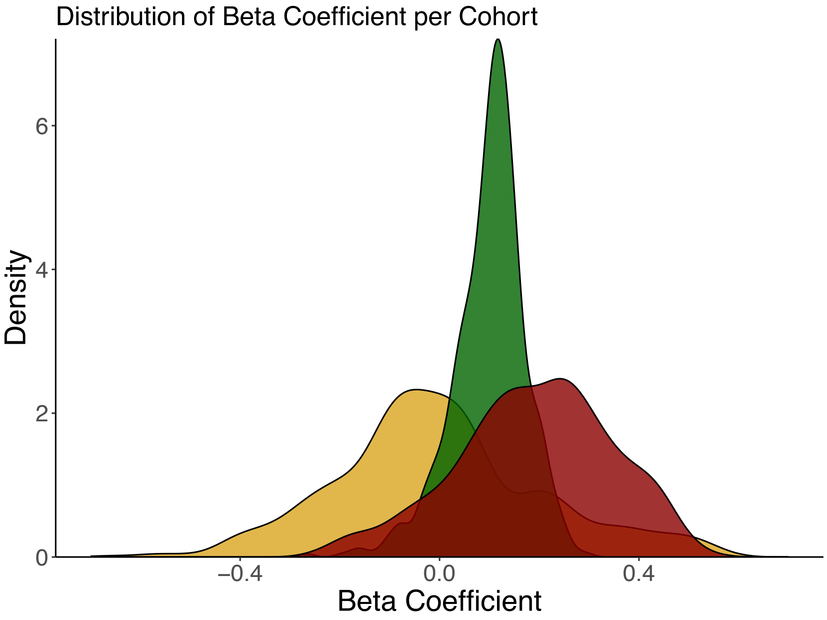
Distribution of Beta Coefficient per Cohort
Density
6
4
2
0
−0.4
0.0
0.4
Beta Coefficient

B

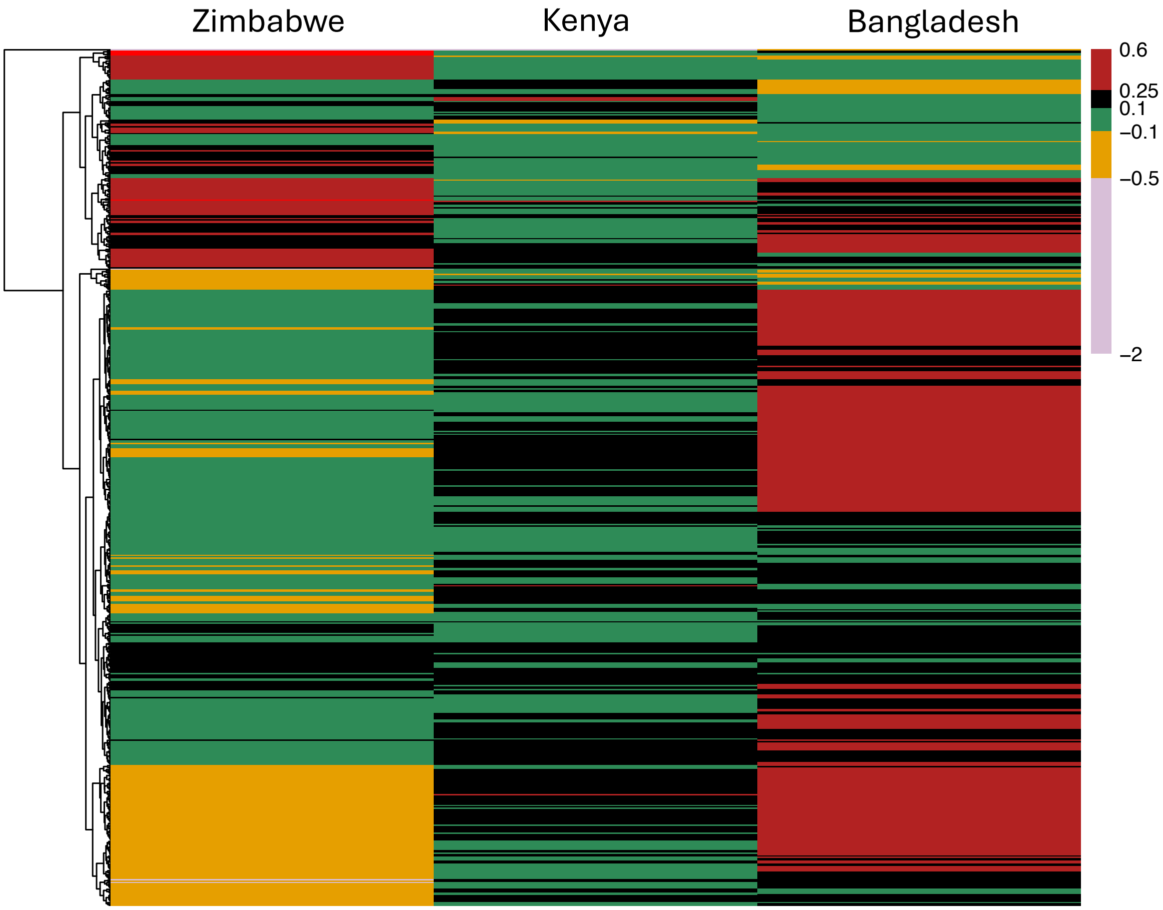

Figure S4. A. Distribution of beta coefficients of the logistic regression of PTB and each of maternal lipids. We observe a shift in support for the three cohorts. **B.** Heatmap showing differences of regression coefficients for each lipid across cohorts.

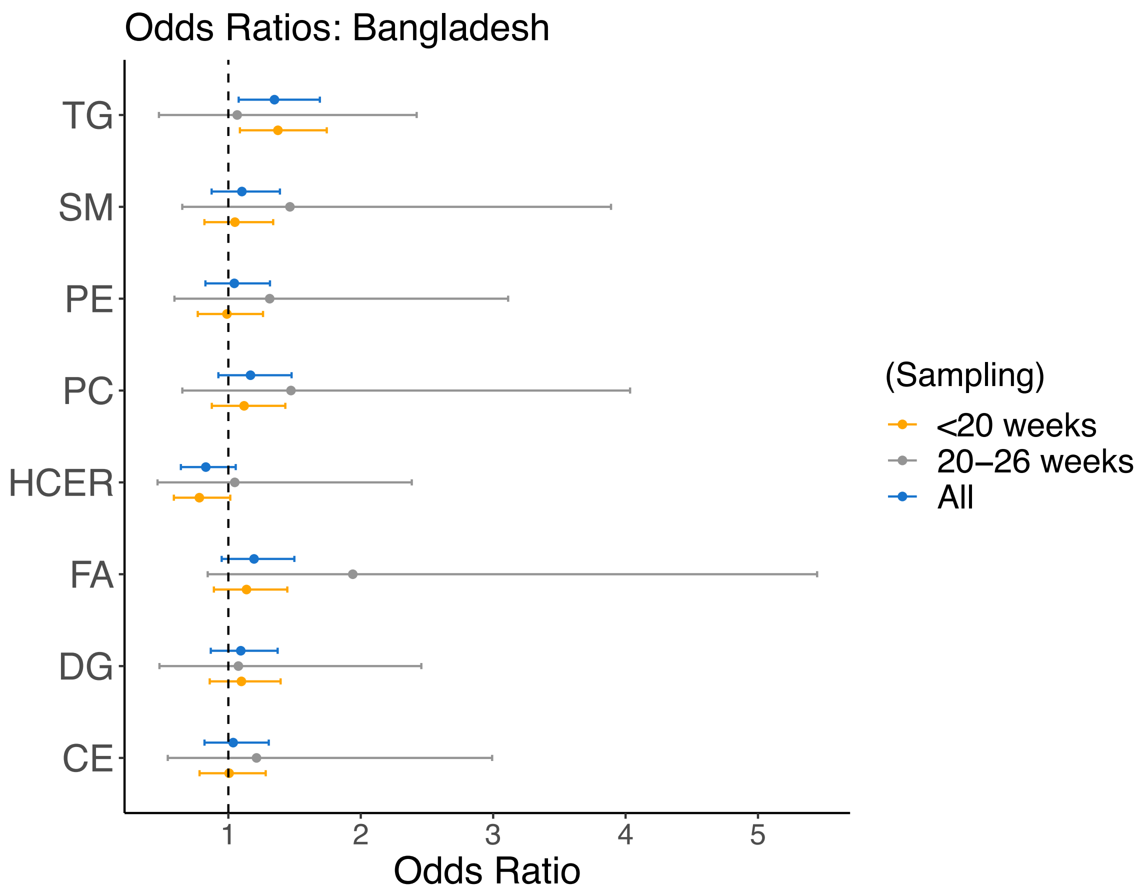

Figure S5. Odds ratios for Lipidome classes and PTB in Bangladesh data.

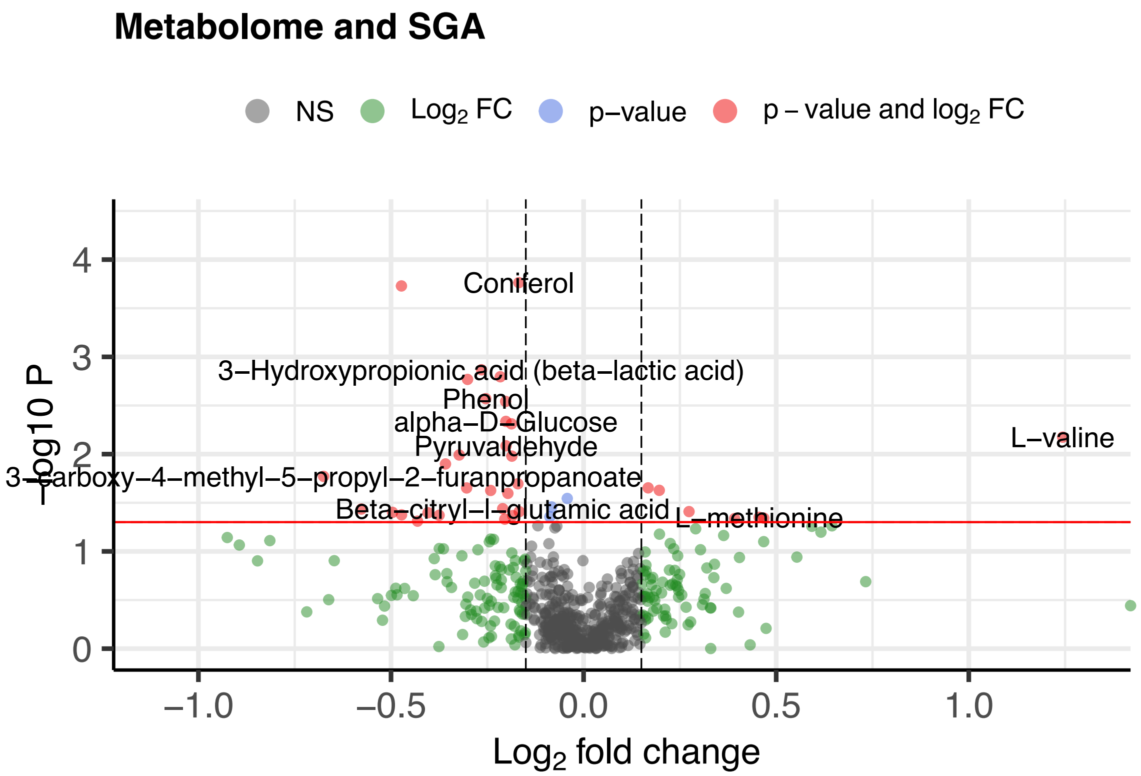

Figure S6. Volcano plots showing association of metabolome with SGA (Intergrowth 21st classification). 39 metabolites had statistically significant changes (p-value<0.05) and none were statistically significant after FDR-adjustment (adjusted p-value < 0.5). Among the 39 metabolites, 74% had decreased levels in SGA.

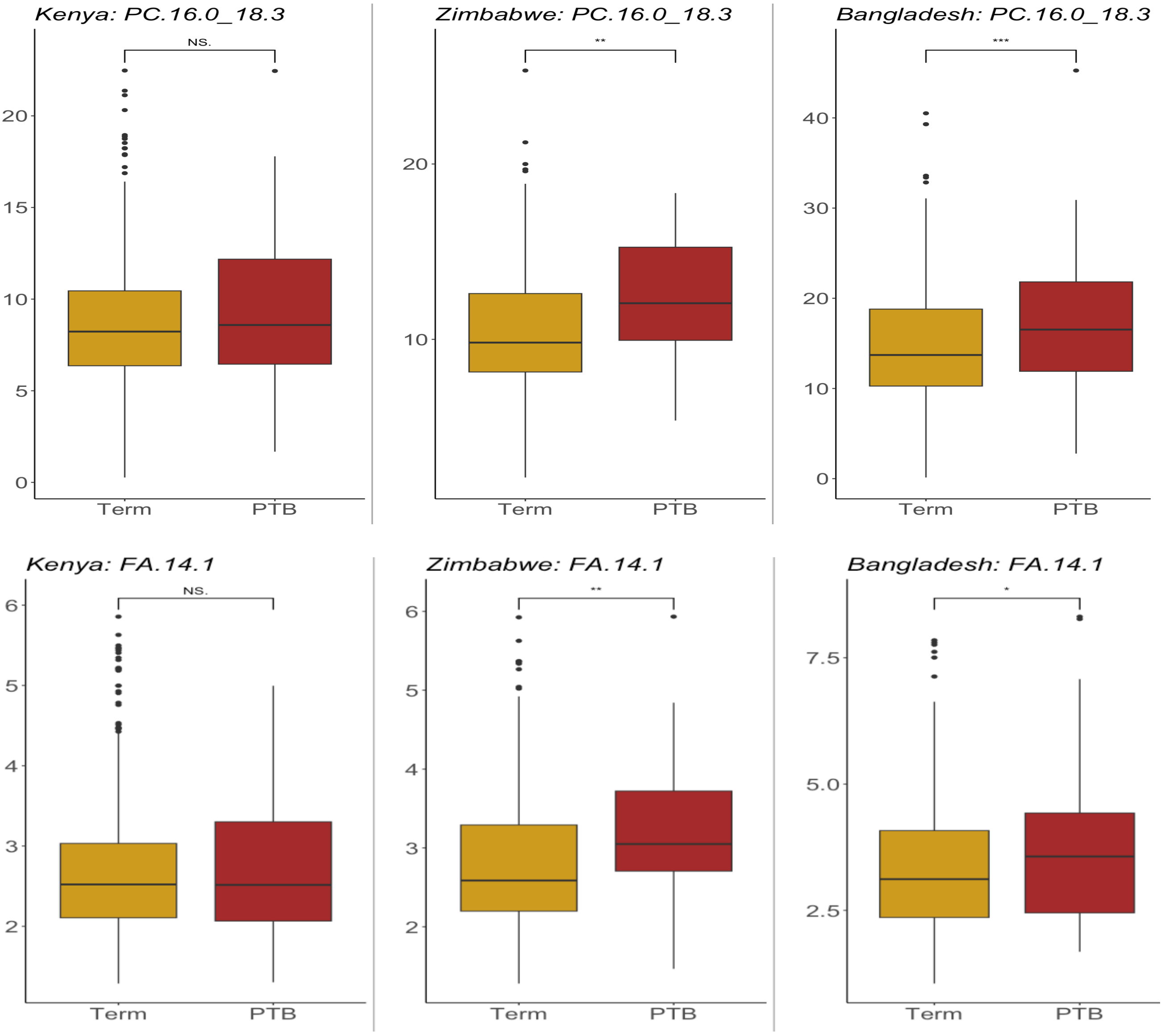

Kenya: PC.16.0_18.3
NS.
Zimbabwe: PC.16.0_18.3
**
Bangladesh: PC.16.0_18.3
***
Kenya: FA.14.1
NS.
Zimbabwe: FA.14.1
**
Bangladesh: FA.14.1
*
Term
PTB

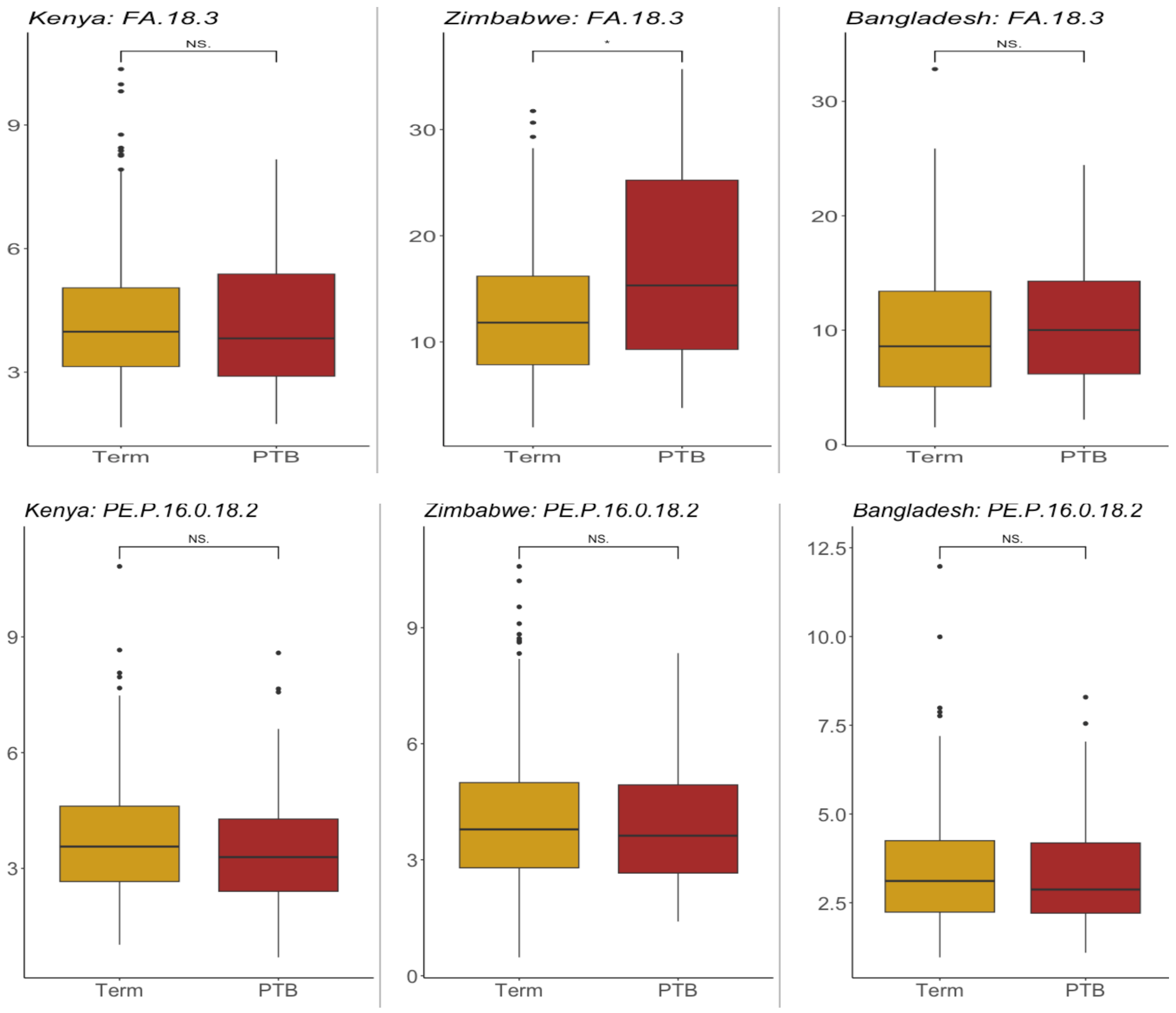
Kenya: FA.18.3
NS.
9
6
3
Term
PTB
Zimbabwe: FA.18.3
*
30
20
10
Term
PTB
Bangladesh: FA.18.3
NS.
30
20
10
0
Term
PTB
Kenya: PE.P.16.0.18.2
NS.
9
6
3
Term
PTB
Zimbabwe: PE.P.16.0.18.2
NS.
9
6
3
0
Term
PTB
Bangladesh: PE.P.16.0.18.2
NS.
12.5
10.0
7.5
5.0
2.5
Term
PTB

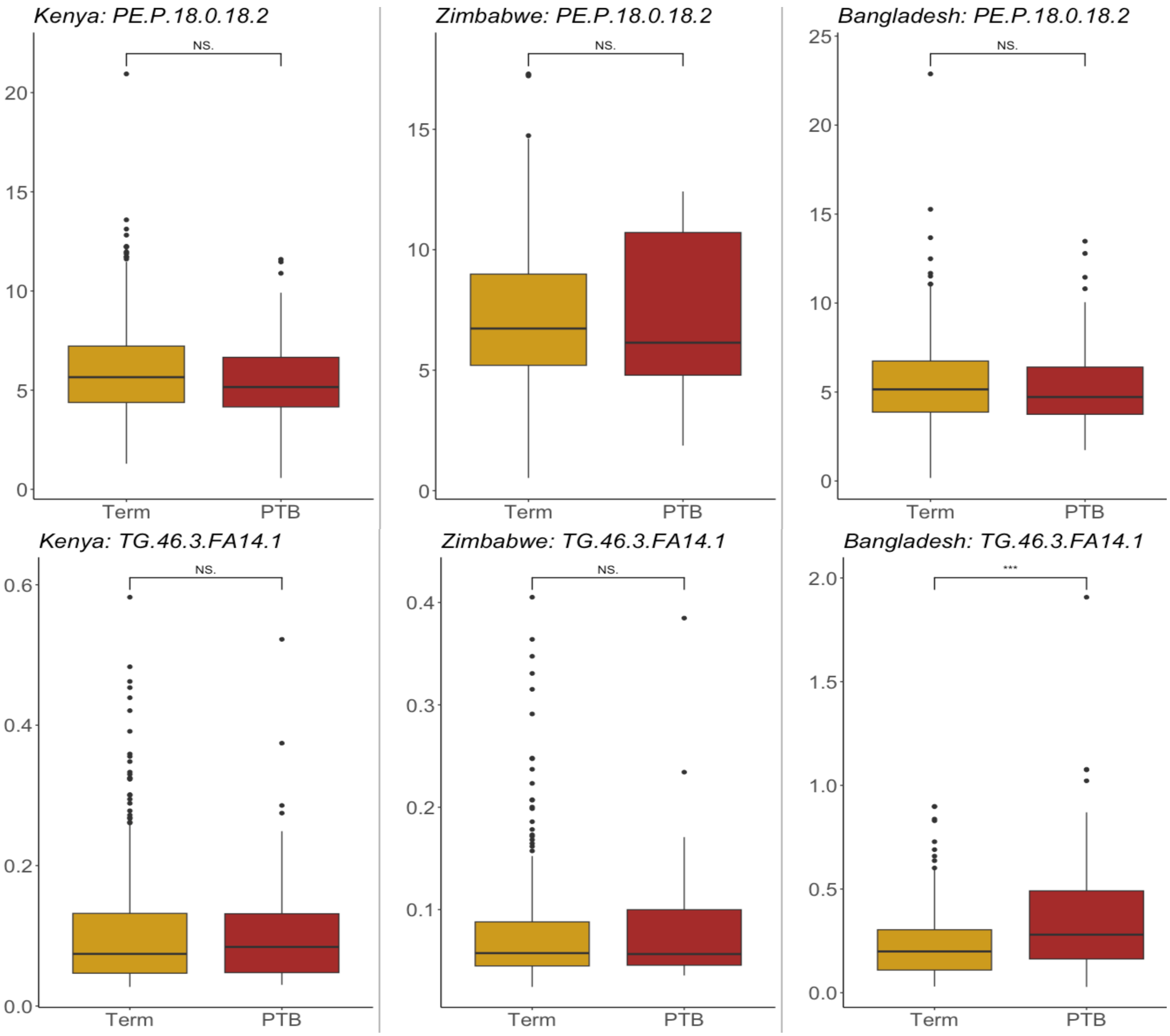

Kenya: PE.P.18.0.18.2
Zimbabwe: PE.P.18.0.18.2
Bangladesh: PE.P.18.0.18.2
Kenya: TG.46.3.FA14.1
Zimbabwe: TG.46.3.FA14.1
Bangladesh: TG.46.3.FA14.1
NS.
***
Term
PTB

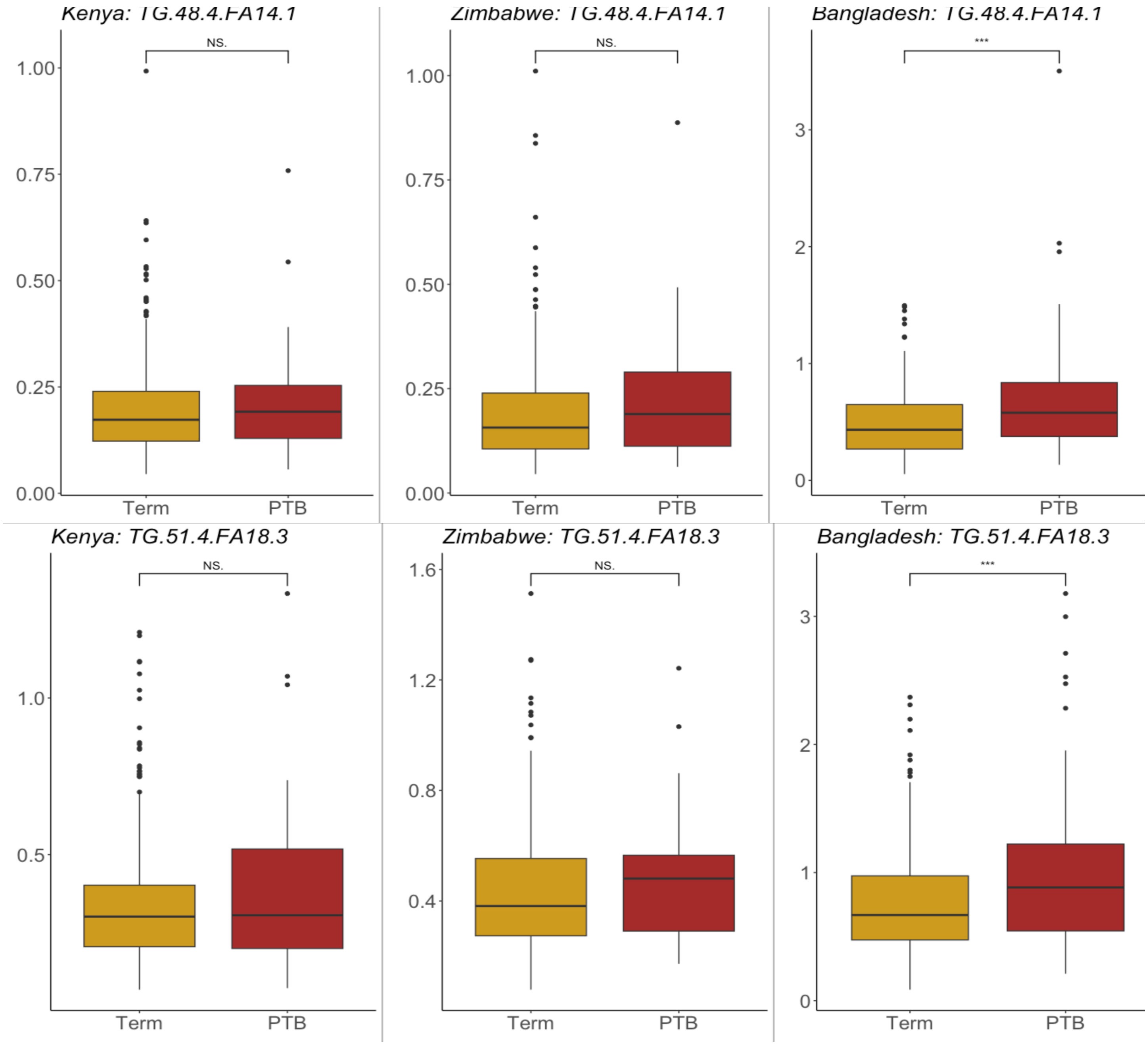

Figure S7. Eight biomarkers identified by ICP and their values across cohorts. We observe constant trends for each cohort.
